# Effects of telemetry collars on two free-roaming feral equid species

**DOI:** 10.1371/journal.pone.0303312

**Published:** 2024-05-30

**Authors:** Kathryn A. Schoenecker, Sarah R. B. King, Jacob D. Hennig, Mary J. Cole, J. Derek Scasta, Jeffrey L. Beck

**Affiliations:** 1 U.S. Geological Survey, Fort Collins Science Center, Fort Collins, CO, United States of America; 2 Colorado State University, Fort Collins, CO, United States of America; 3 University of Wyoming, Laramie, WY, United States of America; 4 University of Arizona, Tucson, AZ, United States of America; University of Ferrara Department of Life Sciences and Biotechnology: Universita degli Studi di Ferrara Dipartimento di Scienze della Vita e Biotecnologie, ITALY

## Abstract

There are two species of free-roaming feral equids in North America: horses (*Equus caballus*) and donkeys or “burros” (*E*. *asinus*). Both species were introduced as domestic animals to North America in the early 1500s and currently inhabit rangelands across the western United States, Canada, and all continents except Antarctica. Despite their global distribution, little is known about their fine scale spatial ecology. Contemporary research tools to assess space use include global positioning system (GPS) tracking collars, but older models were problematic due to stiff collar belting causing poor fit. We tested modern designs of GPS collars on *n* = 105 horses and *n* = 60 burros for 4 years in five populations (3 horse, 2 burro) across the western United States, to assess whether collars posed welfare risks to horses or burros. We found no difference in survival of collared versus uncollared mares and jennies, and no difference in survival of their foals. In 4036 of 4307 observations for horses (93.7%) and 2115 of 2258 observations for burros (93.6%), collars were observed symmetrical, maintaining proper fit on the neck. Fur effects from collars (sweaty neck, indented fur, broken fur) were seen in 3% of horse observations and 25% of burro observations. Superficial effects (chafes and marks on skin surface) were seen in 2% of horse observations and 11% of burro observations; no severe effects from collars were seen. Body condition was not affected by collars; mean body condition of collared horses was 4.70 ± 0.54 (mean ± s.d) and 4.71 ± 0.65 for collared burros. Behavior results indicated minimal effects; collared horses stood slightly more than uncollared, and collared burros stood and foraged more in one population, but not in the other. For 6.3% of observations of horses and 6.4% of observations of burros, we found an effect of time wearing a collar on the cumulative sum of fur effects which increased over time (burros: r_s_ = 0.87, P = <0.0001; horses: r_s_ = 0.31, P = 0.002). Burros also showed an increase over time in the number of superficial effects, but horses did not. Collars occasionally moved into the wrong position, shifting forward over the ears; we observed this on 19 horses and 1 burro. Of those, most collars went over the ears in summer (*n* = 12). All collars were equipped with a remote release mechanism as well as a timed-release mechanism for redundancy, thus removed when observed in wrong position to avoid rubbing or discomfort. Our finding of no consequential physical effects in 98% of horse observations, and 89% of burro observations suggests the consequences of collars on free-roaming equid welfare and survival is biologically insignificant, although collars should be monitored regularly and continue to be equipped with a remote release mechanism to remove a collar if needed. With frequent welfare-driven, visual monitoring, collaring of free-roaming equids can be a safe and useful tool to increase our understanding of their spatial ecology, demography, habitat use, behavior, and interactions with other wildlife.

## Introduction

In wildlife research, the welfare and safety of the study species is paramount [1–3]. This principle is not only important for the integrity of the wildlife profession, but also critical for upholding the public trust [4, 5]. Feral species have a unique relationship with humans due to their history of domestication [6, 7] and can elicit strong emotion and opinions about their management. Thus, the same principle applies to free-ranging feral species in which study techniques need to be safe and humane. This is particularly true for feral equids that enjoy considerable esteem with the public [8] and are also a protected species in some populations in the United States.

There are two species of free-roaming feral equids in North America. The current free-roaming horse (*Equus caballus*) was originally domesticated in Eurasia over 5,500 years ago [6] and introduced to the Americas by Spanish colonists in the 1490s. Free-roaming donkeys or “burros” (*E*. *asinus*) were domesticated from African wild asses (*E*. *africanus*) approximately 7,000–8,000 years ago [7] and brought to North America about the same time as domestic horses. In what is today the United States, introduced horses were skillfully used by native North American peoples for almost 200 years before European settlers arrived [9]. The relationship of these equids with humans shifted to a more utilitarian role after European colonization in the 18^th^ and 19^th^ centuries [9]. Similarly, domestic burros were crucial for draft power and agriculture in South, Central, and North America after their introduction [10]. Eventually, owned domestic horses and burros were released or escaped to the wild becoming feral [11]. Today free-roaming descendants of these domesticated equids inhabit public, private, and tribal lands across the western United States [12, 13], and occur in many other countries on every continent except Antarctica [14]. In the United States, the U.S. Congress passed legislation to protect horses and burros on some federally managed public lands under the Wild Free-Roaming Horses and Burros Act of 1971 (Public Law 92–195) because of their “iconic status as a cultural legacy” over thousands of years of interaction with human cultures.

Free-roaming horses and burros inhabit considerable expanses of rangelands across the western United States and Canada but relatively little is understood about their fine scale spatial ecology and other population attributes, which have been quantified relatively imprecisely. This is particularly true compared to the extensive published literature on the biology, life cycle, population dynamics, spatial ecology, seasonal movements, migrations, ecosystem function, and role of native North American ungulates [e.g., 15–18] that have been equipped with VHF or GPS collars since the 1950s. In 2013, the National Research Council advised more research was needed on several topics related to horse and burro management, including increased information on basic horse and burro ecology [19]. Since then, many studies have added to the body of knowledge on free-roaming equid ecology in North America [e.g., 10, 20–23], diet [24, 25], behavior [26, 27], and interactions with native wildlife [e.g., 28–31]. Yet only a few studies have applied global positioning system (GPS) collar technology in studies of free-roaming equids in North America [10, 20, 32, 33]. This is primarily because in a study conducted in the 1980s, collars caused some severe effects to individual horses, largely due to changes in neck sizes of stallions resulting from abnormally large weight gains from year 1 to year 2 of that study and the lack of a release mechanism on those collars [34]. This raised concerns about the safety of collar applications on free-roaming horses. Further, in a broad review of studies that affixed GPS collars on equid species globally, authors determined that most studies do not provide sufficient information to assess relative risk of collar-related complications and recommended explicit reporting and discussion of telemetry collar impacts, particularly on equids, to improve understanding of how telemetry collars can affect study individuals [35].

GPS telemetry collars have been deployed on a variety of ungulate species [e.g., 36–42] with success. But in some studies effects from telemetry collars have been reported, including neck lesions in mule deer (*Odocoileus hemionus*) and bighorn sheep (*Ovis canadensis*) [43], decreased movement rates in plains zebra (*E*. *quagga*) [44], declining body condition in mule deer [45], reduced neonate survival in mountain goats (*Oreamnos americanus***)** and moose (*Alces alces*) [46, 47], increased head shaking in Scimitar-horned oryx (*Oryx dammah*) [48], and decreased adult survival in caribou (*Rangifer tarandus*) [49]. However, a recent study found no negative effects from GPS collars on free-roaming horse mares at the Sheldon National Wildlife Refuge, Nevada, USA after 3 years of collar wear [50].

Since the 1980s there have been many advances and improvements in belting material, fit and shape, weight, drop off mechanisms, and communication systems for telemetry tracking collars. In view of the detailed information on free-roaming horse and burro spatial ecology that could be obtained from GPS telemetry technology, we investigated placing GPS collars on free-roaming equids in a comprehensive field study that assessed any welfare, demographic, or behavioral effects. We tested effects of GPS collars on horses and burros in five populations for up to 4 years across the western United States. Our objectives were to assess whether collars posed risks to horse and burro welfare by determining if collars caused no physical effects, insignificant effects, superficial effects (on the skin surface), or severe effects to the necks of horses and burros; whether collars affected behavior or body condition of horses and burros; and if collars affected survival of mares, jennies, or their associated foals. Finally, we tested the success rate of remotely triggered drop-off mechanisms, and evaluated the necks of horses after collars were removed.

## Materials and methods

### Study area

**Fig 1:**
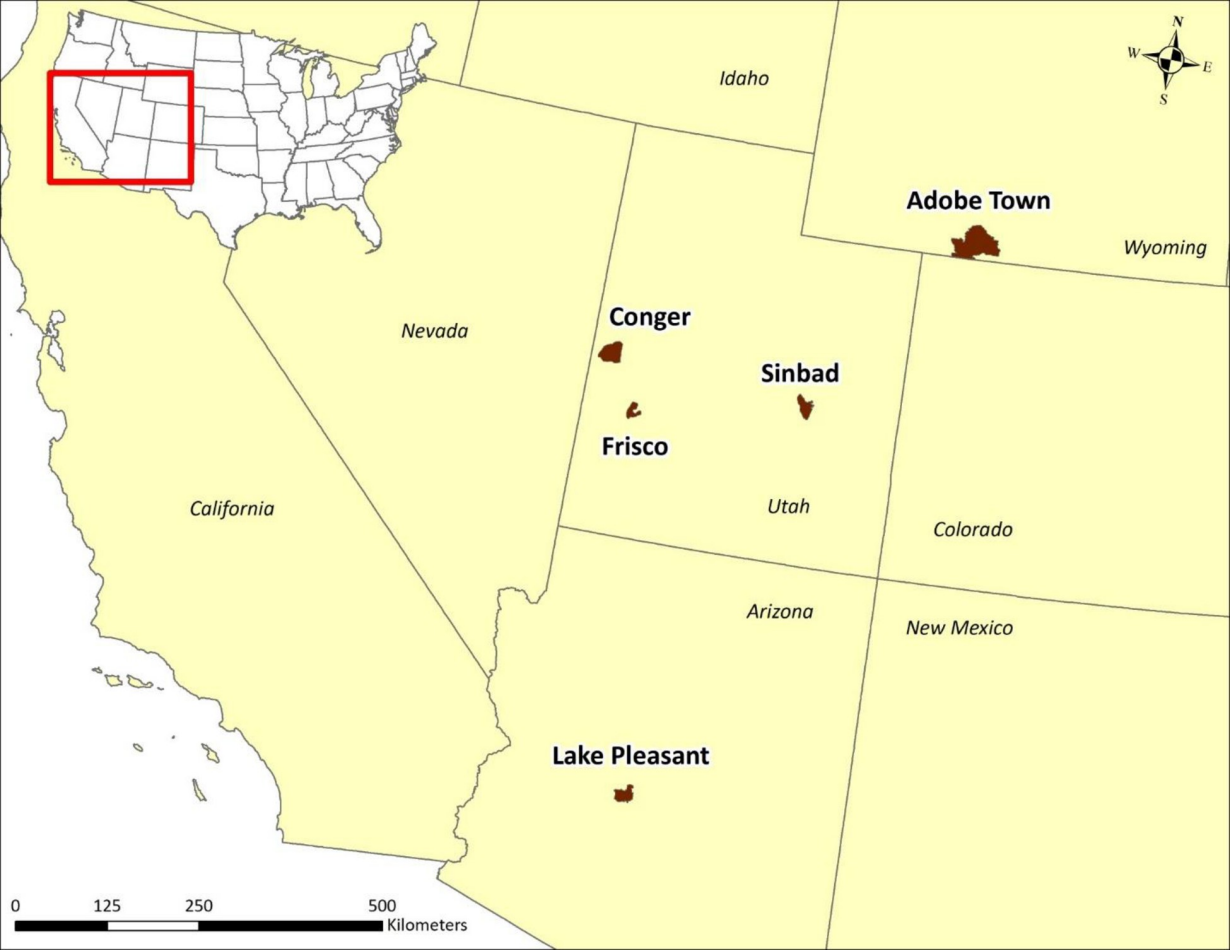
Study area map showing locations of five herd management areas (HMAs) across the western United States, in which we evaluated effects of GPS collars on free-roaming horses and burros, 2016–2020. The Department of Interior, Bureau of Land Management manages each HMA for either horses or burros; HMA polygons are shown in brown along with labels, and red outline shows map area within the United States. HMAs with horses included Adobe Town, Wyoming, and Conger and Frisco, Utah. HMAs with burros were Lake Pleasant, Arizona, and Sinbad, Utah.

We studied 5 free-roaming equid populations in the western United States (3 horse and 2 burro herds; Fig 1). Horses and burros in our study areas were managed by the Department of Interior, Bureau of Land Management (BLM) within designated Herd Management Areas (HMAs) that were also managed for human recreation (camping, hiking, hunting), wildlife conservation, livestock grazing, and other required and permitted uses. None of the HMAs were fully fenced but roads and highways along HMA boundaries may have functioned as semi- or non-permeable borders.

#### Adobe town herd management area

Adobe Town HMA encompasses 195,000 ha, in Wyoming, USA (Fig 1), and ranges in elevation from 1,883–2,506 m [51]. Mean annual precipitation was 277 mm and mean 30-year normal temperature was 6.0°C [52]. The estimated horse population size ranged from 767 to 994 during the study years [53]. Other ungulates in the study area included pronghorn (*Antilocapra americana*), mule deer, elk (*Cervus canadensis*), cattle (*Bos taurus*) and domestic sheep (*Ovi*s *aries*).

Adobe Town is classified as cold-arid-steppe [54]. Dominant shrubs were Wyoming big sagebrush (*Artemisia tridentata wyomingensis*), yellow rabbitbrush (*Chrysothamnus viscidiflorus*), rubber rabbitbrush (*Ericameria nauseosa*), greasewood (*Sarcobatus vermiculatus*), and assorted saltbush (*Atriplex* spp.) species. Perennial grass species included squirreltail (*Elymus elymoides*), inland saltgrass (*Distichlis spicata*), prairie Junegrass (*Koeleria macrantha*), sandhill muhly (*Muhlenbergia pungens*), Sandberg bluegrass (*Poa secunda*), and an annual species, cheatgrass (*Bromus tectorum*).

#### Conger herd management area

Conger HMA encompasses 69,198 ha, in Utah, USA (Fig 1), and ranges in elevation from 1,433 m to 2,478 m. Mean annual precipitation between 2016 and 2020 was 192.15 ± 60.27 mm and average daily mean temperature was 1° C ± 2° C in winter and 27° C ± 2° C in summer (Remote Automatic Weather Station [RAWS] Tule Valley—Delta 49W Utah). The horse population was approximately 100 individuals at the start of the study. Other ungulates in the area included pronghorn, mule deer, and occasionally elk, with cattle at the south end and domestic sheep at the north end during winter.

Conger HMA encompasses the Conger Mountain Wilderness Study Area, characterized by a series of north-south rocky ridges lined with cliffs to the east of Conger Mountain. Higher elevations had two-needle pinyon (*Pinus edulis*) and juniper (*Juniperus* spp.) forest, interspersed with curl-leaf mountain mahogany (*Cercocarpus ledifolius*) and an understory of big sagebrush (*Artemisia* spp.), yucca (*Yucca* spp.), and bunchgrasses (including Idaho fescue [*Festuca idahoensis*], needle-and-thread [*Hesperostipa comata*], basin wildrye [*Leymus cinereus*] muttongrass [*Poa fendleriana*], and bluebunch wheatgrass [*Pseudoroegneria spicata*]). Lower elevations were dominated by sagebrush, with ephedra (*Ephedra* spp.), saltbush, and an herbaceous layer of perennial bunchgrasses such as needlegrass (*Achnatherum* spp.), squirreltail, and Sandberg bluegrass, and an annual species, cheatgrass. Valley bottoms had salt desert scrub and semi-desert shrub, with saltbush, sagebrush, various forb and perennial and annual graminoids such as Indian ricegrass (*Achnatherum hymenoides*), blue grama (*Bouteloua gracilis*), thickspike wheatgrass (*E*. *lanceolatus*), western wheatgrass (*Pascopyrum smithii*), bluegrass (*Poa* spp.), and cheatgrass.

#### Frisco herd management area

Frisco HMA encompasses 24,429 ha, in Utah, USA (Fig 1), and ranges in elevation from 1,700 m to 2,895 m. Mean annual precipitation between 2017 and 2020 was 177.86 ±104.62 mm and average daily mean temperature was 0 ± 2° C in winter and 24 ± 2° C in summer (RAWS Brimstone Res.–Milford 20WSWS Utah). The horse population was approximately 100 individuals at the start of the study. Other ungulates in the area included pronghorn, mule deer and elk, with cattle present in winter.

Frisco HMA includes Frisco Peak in the San Francisco Mountains and the lower ridge of the Beaver Mountains. The highest elevations near Frisco Peak had spruce (*Picea* spp.)–subalpine fir (*Abies lasiocarpa*) forest, with remaining upper elevations covered in Great Basin pinyon–juniper woodland. There were areas of cliffs, scree slopes, and rocky outcrops. In addition to pinyon pine and juniper, these areas had some ponderosa pine (*Pinus ponderosa*), aspen (*Populus tremuloides*), Gambel oak (*Quercus gambelii*), and shrubs such as sagebrush and mountain mahogany (*Cercocarpus* spp.). The herbaceous layer consisted of bunchgrasses and cheatgrass like Conger HMA. Lower elevations were dominated by sagebrush shrubland with patches of juniper and sparse grasses such as needlegrass, blue grama, thickspike wheatgrass, fescue, needle-and-thread (*Hesperostipa comata*), basin wildrye, western wheatgrass, bluegrass, and cheatgrass. Valley bottoms were semi-desert shrub steppe, characterized by grasses such as Indian ricegrass, blue grama, saltgrass (*Distichlis spicata*), saline wildrye (*L*. *salinus*), bluegrass, James’ galleta (*Pleuraphis jamesii*), alkali sacaton (*Sporobolus airoides*), needle-and-thread, and cheatgrass, with shrubs including saltbush, sagebrush, rabbitbrush (*Chrysothamnus* spp.), and winterfat (*Krascheninnikovia lanata*).

#### Lake pleasant herd management area

The Lake Pleasant HMA encompasses 41,900 ha, in the Sonoran Desert, Arizona, USA (Fig 1), and ranges in elevation from 427 to 1174 m. Mean annual precipitation was 323.1 ± 127.1 mm, mostly monsoonal from July to September, and mean monthly temperature varied between 12.6 ± 2.1°C in December and 33.2 ± 0.2°C in July (PRISM Time Series Data: Period: 2017–01–2019–12; location: Lat: 33.9576, Lon: -112.2863, Elevation: 564m; S1 Fig). The burro population was approximately 600 at the start of the study (many of which were located outside HMA boundaries). Other ungulates in the area included desert mule deer (*O*. *h*. *eremicus*) and collared peccary (*Tayassu tajacu*), with low-density cattle present year-round.

The Sonoran Desert is considered the most tropical North American desert in which the climate is frost-free and summer monsoons originate in tropical oceans [55]. It has biogeographical similarities with more tropical ecological communities and vegetation differs from those of the shrub-dominated Chihuahuan, Great Basin, and Mojave deserts of North America [55]. Vegetation consisted of succulents such as saguaro (*Carnegiea gigantea*), cholla (*Cylindropuntia* spp.), ocotillo (*Fouquieria splendens*), and prickly pear (*Opuntia phaeacantha*), with acacia (*Acacia* spp.), creosote bush (*Larrea tridentata*), tamarisk (*Tamarix* spp.), and leguminous trees such as palo verde (*Parkinsonia spp*.), and mesquite (*Prosopis* spp.), providing shrub and tree cover. Herbaceous vegetation in the understory included grama (*Bouteloua* spp.), Arizona cottontop (*Digitaria californica*), curly mesquite grass (*Hilaria belangeri*), little barley (*Hordeum pusillum*), big galleta (*Pleuraphis rigida*), Bigelow bluegrass (*Poa bigelovii*), sixweeks fescue (*Vulpia octoflora*), and panic grasses (e.g., *Brachiaria arizonica* and *Panicum hirticaule*) [55].

#### Sinbad herd management area

Sinbad HMA encompasses 40,200 ha on the San Rafael Swell of the Colorado Plateau in Utah, USA (Fig 1) and ranges in elevation from 1295 m to 2082 m. Mean annual precipitation was 254.2 ± 34.9 mm (range: 5.1 ± 4.9 mm in June to 34.8 ± 25.6 mm in January) and mean monthly temperature was -1.7 ± 1.6°C in winter and 24.5 ± 0.5°C in summer (PRISM Time Series Data: Period: 2016–01–2019–12; Location: Lat: 38.9054, Lon: -110.5469, Elevation: 1939 m; S1 Fig). The burro population was approximately 100 individuals at the start of the study. Other ungulates in the area included pronghorn and bighorn sheep, with cattle present in winter.

Sinbad HMA is comprised of canyonlands and mesas interspersed with open valleys. Vegetation was primarily juniper shrubland with meadow grasslands. The area was dominated by stands of juniper and two-needle pinyon pine, with shrubs including sagebrush, yellow rabbitbrush, ephedra, and Spanish bayonet (*Yucca harrimaniae*), and an understory of herbaceous plants including needle and thread, Indian ricegrass, James’ galleta (*Hilaria jamesii*), and *Astragalus* spp. [56].

### Methods

#### Animal handling and radio collar deployment

The Bureau of Land Management conducted management gathers (capture and removal) by helicopter or bait trapping in each HMA at the start of the study: Adobe Town horses (Feb/Mar and Oct 2017), Conger horses (Aug 2016), Frisco horses (Feb 2017), Lake Pleasant burros (Feb 2017–Jun 2018; trapping) and Sinbad burros (Apr 2015), following approved protocols [57]. The BLM returned animals to the range, so each population had approximately 100 animals representing a 50:50 sex ratio and ages foal-to-16+ years old, with the exception of Adobe Town which had ~800 horses and Lake Pleasant which had ~600 burros. In each population, equids inhabited areas within and sometimes outside of HMA boundaries.

Telemetry collars were fitted on horses and burros following methods described in S1 Text and a previous captive study [58] and were affixed by the same individuals (K. Schoenecker and S. King) in all 5 populations for consistency. Each collar was equipped with a timed release drop off and a remote release drop-off mechanism to facilitate immediate collar removal during the study: a redundancy in safety that was required by the management agency. For collar fitting, horses and burros from Adobe Town, Conger, Frisco, and Sinbad HMAs were loosely restrained in a padded hydraulic squeeze chute. For Lake Pleasant burros and some Adobe Town horses we used a transportable squeeze chute in the field to loosely restrain them during collaring. Collars were placed high on the neck and fitted so they were not too tight or loose (S1 Text), and all animals were observed in corrals after 24–48 hours to assess whether collar fit appeared correct (e.g., collars not moving up or down the neck excessively) and adjusted if needed. We aged all individuals by examining tooth eruption and wear [59], recorded pelage characteristics, photographed each individual, and except for Adobe Town horses, freeze marked them on the left hip with an identifying number.

Collars were placed on adult female (≥3 years old) burros and horses with Henneke body condition scores ≥4 [60]. Each HMA had a minimum of 30 individuals (Table 1) collared with either Vectronic (Vectronic Aerospace GmbH, Berlin, Germany) or Lotek (Lotek Wireless, Newmarket Canada) iridium GPS collars, or Sirtrack (formerly Havelock North, New Zealand) very high frequency (VHF) radio collars. Collars were either oval or teardrop in shape, based on results from a previous study [58]. All research was approved by an Institutional Animal Care and Use Committee protocol (# FORT-IACUC 2015–10) through the U.S. Geological Survey, Fort Collins Science Center, Fort Collins, Colorado, and a Colorado State University Inter-Institutional Agreement (ACUC concurrence). At Adobe Town HMA, research was approved by and followed University of Wyoming Institutional Animal Care and Use Committee protocols 20160826DS00249‐01 and 20190802DS00385‐01.

**Table 1:** Study areas, sample sizes, dates of observations, and mean distance to horses and burros wearing radio collars from 2016 to 2020 in a study measuring potential effects of GPS collars on free-ranging feral equids in 5 BLM herd management areas in the western United States (WY = Wyoming; UT = Utah; AZ = Arizona).

Species	Study area	Date range collared females were observed	Number of collared females	Number of collared females in behavioral observations (total hours of observations)	Number of collar check observations	Mean ± SD distance to animal (m) during collar check observations
**Horse**	Adobe Town, WY	3/4/17-9/21/19	37	None	281	417 ± 344
	Conger, UT	9/7/16-9/20/20	38	24 (1,538 hours)	2414	546 ± 495
	Frisco, UT	3/24/17-9/17/20	30	None	1612	464 ± 571
**Burro**	Lake Pleasant, AZ	4/4/17-4/19/20	30	23 (1,694 hours)	802	95 ± 136
	Sinbad, UT	4/27/16-9/21/19	30	24 (2,050)	1456	222 ± 365

#### GPS collar monitoring

Every female horse and burro wearing a collar was visually located 1–2 times per month to record data throughout the calendar year for all 4 years of the study, except for horses at Adobe Town which wore collars for 2 years and were located monthly, or as often as possible given access, weather, and time constraints [61]. Once located, we recorded date, time, location, and details of all individuals in the social group including the presence of new foals. We recorded distance from the observer to the animal using the scale bar in the GPS unit (Garmin International, Olathe, Kansas), by estimating distance to topographic features near the group. We recorded body condition of collared individuals using a visual scale in the Henneke score [60] which scales from 1 (emaciated) to 9 (obese). We used binoculars (Zeiss/Nikon/Canon/Vortex, 10 × 40) or a spotting scope (Bushnell/Vortex 20–60 × 80) to observe details of individuals and visual appearance of the collar and the neck of the individual. We looked for any physical effects that could be attributable to the collars and categorized them into categories of “fur” effects (indented fur, sweat visible under the collar, broken fur) or “superficial” effects (chafing or chafe marks [i.e., fur worn away to the skin], sores or wounds, or presence of healed sores [i.e., scabs]). More than one category of wear could be recorded for each animal, and we recorded additional notes such as the appearance of the collar (loose, snug, crooked). We sought to view both sides of the neck and the gullet (under the throat) during each observation period and recorded which side(s) were seen and if a view of the gullet was obtained. We recorded whether the collar was upside down on the neck (spun) or not hanging symmetrically (crooked) or over the ears. If a collar was observed over the ears, we attempted to remove it at the time it was first observed using the triggerable remote release device attached to each collar. To quantify the thoroughness and quality of our observations we assigned a quality-of-observation score from 1 (a brief observation, and/or from a far distance, and/or only one side of the neck) to 5 (a very good observation, all sides of the face and neck seen and high potential to observe effects). At the end of the study, BLM conducted pre-planned gathers at Conger and Frisco HMAs, during which we closely examined horses’ necks to record any effects after multiple years of wear.

#### Behavior

We recorded behaviors of collared animals and any uncollared associates of horses at Conger HMA and collared and uncollared associate burros at Lake Pleasant and Sinbad HMAs to evaluate if GPS collars influenced or modified behavior (Table 1). We collected behavior data over 4 years; at Conger and Sinbad between March and September annually and at Lake Pleasant year-round, using scan sampling every 5 minutes during an observation hour [62] to record the basic state of individuals in a group (feeding, moving, standing, other). We used the GPS location on collars to determine areas recently frequented by a collared individual, and then found the precise location using the VHF signal. We observed individuals through binoculars or a spotting scope to record data, and remained at a distance sufficient to cause no disturbance to groups or alter their behavior (approximately 100 to 500 m depending on the group). We observed groups throughout the day to represent morning, mid-day, and afternoon behaviors and used systematic sampling to obtain observations in each time period.

#### Foal survival

We examined foal survival to age 1 of foals born to 65 collared horse mares and 59 collared burro jennies, and 33 uncollared mares and 22 uncollared jennies during all years of the study at Conger, Frisco, Sinbad, and Lake Pleasant HMAs. All foals whose survival was known within the first 12 months after first being identified were included. We included foals born in the last year of the study who were observed until the study ended, when foals were 0 to 8 months old.

#### Statistical analyses

Data were collected in the field on paper data sheets and entered into a web-database developed and administered by the U.S Geological Survey, Fort Collins Science Center. We used DBI and RSQLite packages [63, 64] to bring data from the database into the R environment [65] for analyses.

*Analyses of collar effects*. We analyzed data from all five populations to evaluate differences in collar effects across species, sites, and years. To examine collar fit and overall effects, and the relationship between relative distance to collared individual and quality of observations we used all data collected; but for all other analyses we censored data to remove rows where collar effects were unknown or not recorded (i.e., the distance was too great to determine any effects other than to confirm that the collar was not over the ears). We defined seasons as winter: December to February; spring: March to May; summer: June to August; and fall: September to November.

We removed any rows in the physical effects, body condition, observation quality, year, and distance columns if no effects were observed and nothing was recorded, and converted all discrete variables to factors. We log-transformed distance from observer to the animal and removed observations where the observer was >2,000 m from the animal (*n* = 53 of 5,735). We converted calendar year dates to “year-of-study” (a continuous variable of 1 to 4 years) to reflect duration of wearing collars. After testing for collinearity of continuous variables using package ‘GGally’ ([66]; i.e., distance and year of study) we used a correlation cutoff of |*r|* ≤0.7, we assessed variance inflation factor (<3) using package ‘usdm’ [67], we fit generalized linear models for binary data (i.e., whether effects were observed or not) using package ‘lme4’ [68], and then evaluated each physical effect separately. For each model physical effects observed was the response variable, iteratively adding fixed variables: year of study, season, distance, observation quality code, body condition, and sides of the neck observed (no sides reported, one side, both sides). We evaluated model fit by comparing differences in Akaike Information Criterion values for small samples (AIC_c_) [69] due to the small sample size of observed effects relative to the total number of observations.

To explore the relationship between distance from observer to the animal and observation quality code, we evaluated the full dataset for a normal distribution with an Anderson-Darling test [70] and based on those results we used a Kruskal-Wallis [71] test. To examine effects of body condition on collar fit, we computed chi-squared goodness-of-fit tests. After testing for normality with a Shapiro-Wilk test, we used Spearman’s rank correlations (*r*_s_) to examine effects of duration of time wearing a collar on physical effects observed and whether the collar slipped over an animal’s ears.

To assess if there were progressive physical effects in which one effect led to another, we evaluated whether observations with an effect were reported in sequential order of severity, such as sweaty neck, then indented fur, then broken fur or chafe, then wound or scab. Due to small sample size, for analysis we combined hair loss with chafe (‘chafe’) and scab with wound (‘wound’). We defined a progression as a sequential set of observations where an effect was recorded. If multiple effects were recorded during an observation, we retained only the most severe effect relative to an *a priori* hierarchical progression (sweaty neck → indented fur → chafe → wound).

*Analyses of behavioral effects*. To examine effect of collars on frequency of feeding, moving, and standing we included all data from adult females (≥2 years old at the time of observation) that had a minimum of 4 hours of behavioral observations within a study year for horses at Conger and burros at Lake Pleasant and Sinbad HMAs. There were more observations on collared mares (*n* = 24; 1,538 observation hours) than uncollared mares (*n* = 25; 867 observation hours), and uncollared horses were from younger age classes, so we paired age classes of collared and uncollared individuals to control for an effect of age on behavior, to ensure comparisons between collared and uncollared individuals were from similar age classes (2–10 years old or classed as ‘adult’ when exact age was not known) resulting in *n* = 17 collared mares with 877 observation hours, and *n* = 25 uncollared mares with 867 observation hours for comparative analyses. At Lake Pleasant there were more observation hours on collared burros (1,694 hours) than uncollared (1,287 hours), but more uncollared individuals than collared observed for behavior data collection (*n* = 23 collared jennies; *n* = 66 uncollared jennies), so we used all data from Lake Pleasant for analysis. At Sinbad we recorded data on 24 collared burros (2,050 observation hours) and 38 uncollared burros (838 observation hours). Uncollared burros at Sinbad were mostly younger adults (≤8 years old), thus we paired age classes of collared and uncollared individuals to control for an effect of age on behavior, including collared jennies 2–7 years old or those classed as “adult’ if exact age was not known, resulting in a sample size of *n* = 19 collared burros (1,269 observation hours) and *n* = 38 uncollared burros (837 observation hours). We fit generalized linear mixed models using the R package ‘lme4’ [68] and tested for overdispersion using package ‘RVAideMemoire’ [72]. We modeled the frequency of each behavior (feeding, standing, or moving) per hour as the response variable, with year as a fixed effect using a binomial distribution with a logit link function. We modeled each study area separately and included a random effect of study individual to account for repeated observations of the same individuals across years. We evaluated univariate, additive, and interaction combinations of collar status (collared or uncollared) and year and used AIC_c_ to rank models. We calculated 95% Wald confidence intervals for models with the lowest AIC_c_ score and considered parameters useful for inference if their 95% confidence intervals did not overlap zero.

*Analyses of survival*. To assess adult female survival (i.e., ≥2 years old), we included only females who were at least 2 years old by the end of the study and had a known fate (i.e., confirmed dead or alive). After testing for normality with a Shapiro-Wilk test, we tested for differences in foal survival between collared and uncollared mares and jennies using a paired Student’s t-test on percent of foals surviving to 12 months of age or the end of the study in each study year.

*Analyses of remote drop off success*. We first tested for normality of data with a Shapiro-Wilk test. Most data were not normal, so we analyzed data using non-parametric methods. We used Spearman’s rank correlation tests to explore effect of collar deployment in months on success or failure of remote drop off attempts, and Kruskal-Wallis tests to explore year effects.

## Results

**Fig 2:**
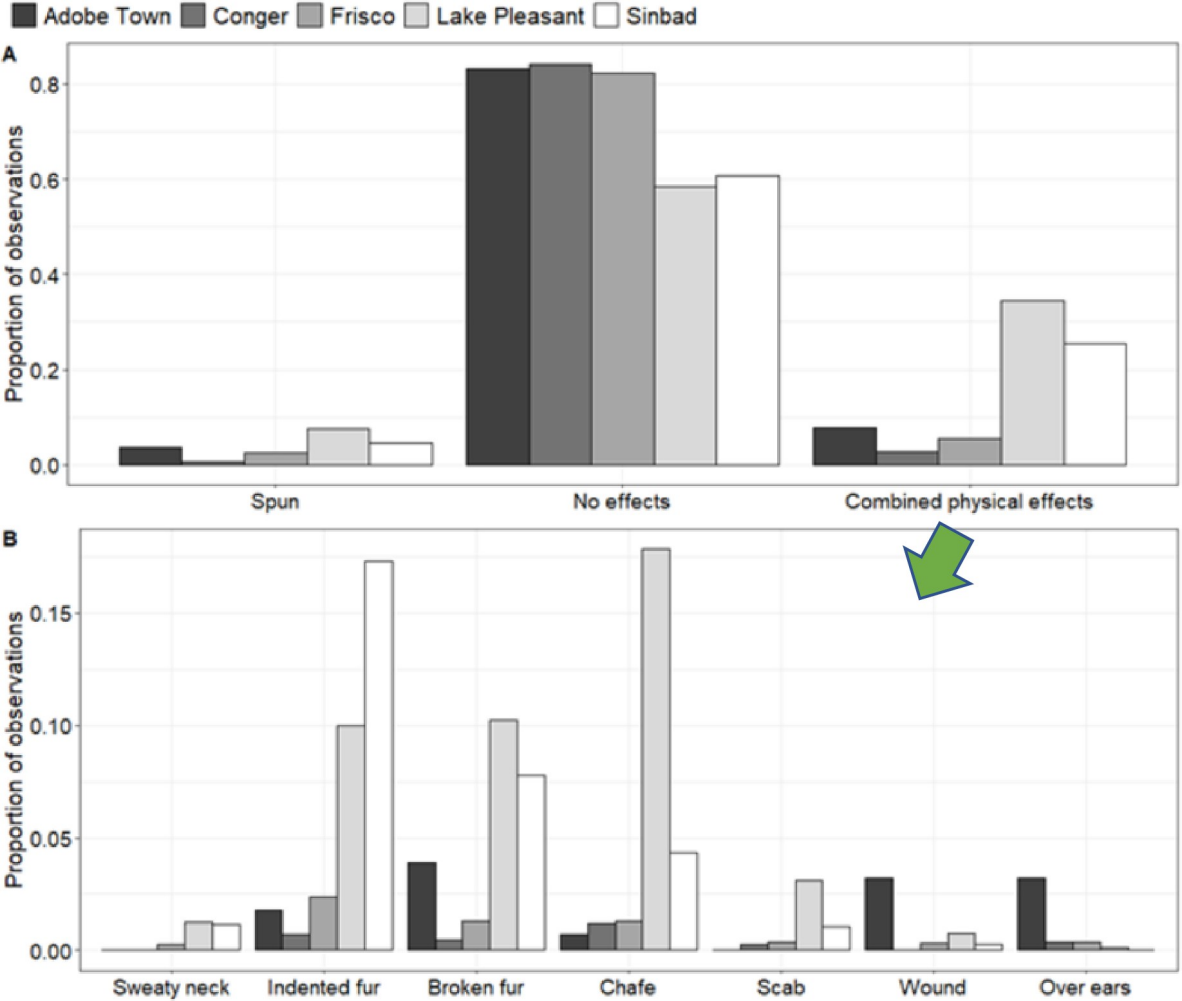
Proportion of effects observed by study area. A. Proportion of observations where collars were observed upside down on the animal’s neck (spun); no effects were observed; or some effects were observed. B. Detailed breakdown of effects from panel A, including the proportion of observations of sweaty neck, indented fur, broken fur, chafe, scab, wound, or collar over ears. Observations were collected between 2016 and 2020, in 5 Herd Management Areas (HMAs) in the western United States. Horse HMAs were Adobe Town, Wyoming; Conger, Utah; Frisco, Utah. Burro HMAs were Lake Pleasant, Arizona and Sinbad, Utah. Although horses at Adobe Town HMA appeared to have a higher proportion of wounds and collar-over-ears, those effects were similar in proportion to other herds; the metric was proportion of effects/observation and Adobe Town had fewer observations than other study areas.

The number of individuals observed and monitored was similar across study sites, although distance to animals observed varied by HMA (Table 1) with the effect of study area being stronger than species (Table 2). Observation quality code was analogous to distance from observer to animal (Kruskal-Wallis F = 2038.7, df = 4, P = <0.0001; Table 2, S1 Fig); burros were observed from closer distances (Table 1). Burros had a higher probability of showing any physical effects from collars than horses (Lake Pleasant: *p* = 0.22, Sinbad: *p* = 0.24; Fig 2), although this was not entirely related to observer distance. Even at the median observation distance (380 m across all study areas) there was a low probability for horses to show any physical effects (Adobe Town: *p* = 0.08; Conger: *p* = 0.03, Frisco: *p* = 0.05; Fig 2). The best model only explained 25% of total variance, likely due to the low number of any physical effects being observed (Table 2). For most observed effects, study area and observation code or distance were the best models (Table 2, S1 Table). Observing just one side of the animal’s neck was ranked higher than seeing both sides, but there was little difference in strength of the model between whether only one or both sides were seen (Table 2 and S1 Table).

**Table 2:** Candidate models, number of parameters (K), ΔAIC_c_, Akaike weight (w_i_), and log-likelihood (LL) to evaluate the role of study area (Adobe Town, Conger, Frisco, Lake Pleasant, or sinbad herd management area), species (horse or burro), distance of observer to collared individual, observation quality score (obs code), body condition (BC), year of study, season (winter, spring, summer, fall), and which side of the animal’s neck was viewed (one side, both sides) on whether any physical effects (i.e., sweaty neck, indented fur, broken fur, chafe, scab, wound, or over the ears) were observed and recorded among feral horses and burros in 5 populations in the western United States between 2016 and 2020, USA.

Model	AICc	K	ΔAIC_c_	w_i_	LL
**Study area + obs code**	3495.06	9	0	1	-1738.51
**Study area + log10(distance)**	3612.74	6	117.68	0	-1800.36
**Study area + one side**	3671.52	6	176.47	0	-1829.75
**Study area + both sides**	3685.26	6	190.21	0	-1836.62
**Study area + year of study**	3719.97	6	224.92	0	-1853.98
**Study area + BC**	3754.72	12	256.66	0	-1865.33
**Study area + season**	3761.99	8	266.93	0	-1872.98
**Study area**	3763.14	5	268.08	0	-1876.56
**Study area + no sides**	3763.52	6	268.46	0	-1875.75
**Species**	3782.25	2	287.20	0	-1889.13
**Obs code**	3895.02	5	399.96	0	-1942.51
**Log10(distance)**	4002.47	2	507.41	0	-1999.23
**Season**	4603.15	4	1108.09	0	-2297.57
**Intercept**	4609.52	1	1114.46	0	-2303.76
**Year of study**	4610.64	2	1115.59	0	-2303.32

Collars were observed to be in a symmetrical position with correct tightness on the neck in 93.6% of all observations (2115 of 2258 observations) for burros, and 93.7% (4036 of 4307 observations) for horses (Table 3). Collars reported as crooked (not symmetrical), too snug, or too loose on the neck (i.e., not fitting correctly) were only reported on 6.4% of observations for burros (*n* = 143) and 6.3% of horse observations (*n* = 271; Table 3). Physical effects that may have been related to fit were only reported on 56 (2.5%) observations for burros and 31 for horses (0.7%) and were only associated with loose fit for horses.

**Table 3:** Observations of the fit of radio collars on the necks of free-roaming feral horses and burros relative to body condition score, in five populations in the western United States, USA. Data were collected between 2016 and 2020; results are pooled by species. Note that collars could be observed not fitting correctly on one observation, then fitting correctly the next time seen; all individuals were observed at least once with a correctly fitting collar and only a small subset were observed on a few occasions with an incorrect fit. Asterix (*) denotes inability to calculate standard deviation with only 2 observations of 2 horses, one of which had no body condition recorded.

Species (total number of observations)	Appearance of collar on neck	Number of times condition observed (% of total observations)	Number of individual horses or burros observed at least once with collar condition (% of n = 60 collared burros and n = 105 collared horses)	Mean (± SD) body condition score at the time collar appearance on neck was recorded
**Burro (n = 2258)**	Crooked	10 (0.4)	5 (8.3)	5.1 ± 0.6
	Loose	107 (4.8)	36 (60.0)	4.5 ± 0.7
	Snug	26 (1.2)	17 (28.3)	4.8 ± 0.7
	Symmetrical fit and correct tightness	2115 (93.7)	60 (100)	4.7 ± 0.6
**Horse (n = 4022)**	Crooked	4 (0.09)	4 (3.8)	4.3 ± 0.6
	Loose	265 (6.15)	69 (65.7)	4.5 ± 0.6
	Snug	2 (0.05)	2 (1.9)	6.0*
	Symmetrical fit and correct tightness	4036 (93.71)	105 (100)	4.7 ± 0.5

**Fig 3:**
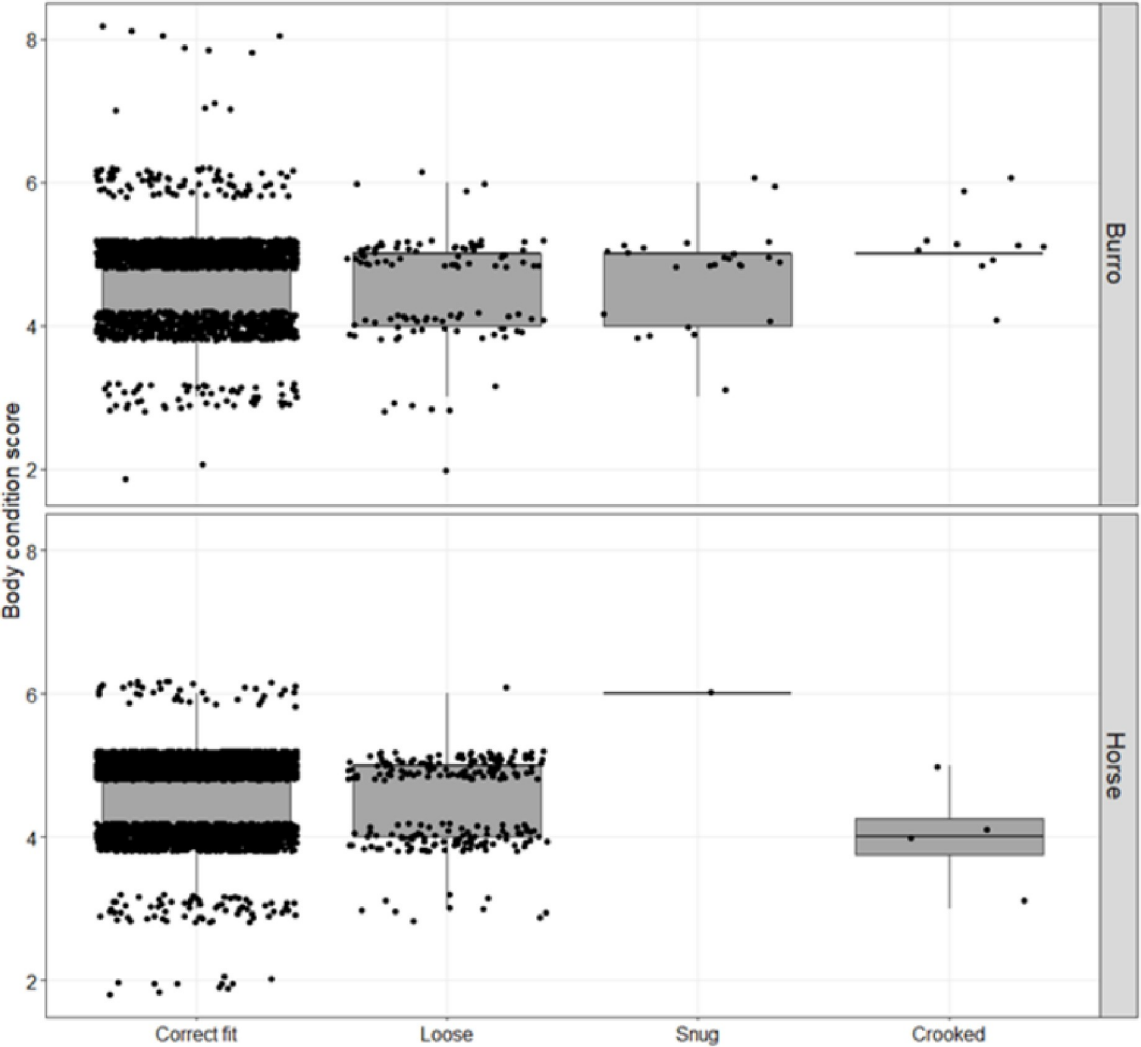
Box plots of body condition score of horses and burros and fit of telemetry collars from five populations of free-roaming feral equids in the western United States, 2016–2020. Body condition scores range from 1 (emaciated) to 9 (obese). Black data points represent each observation in which collar fit and body condition of individual was recorded at the same time. Most collars were observed in correct position and tightness on the necks of horses and burros.

Mean body condition of horses was 4.70 ± 0.54 (mean ± s.d.; range of 2 to 6) and for burros was 4.71 ± 0.65 (range of 2 to 8), meaning in most observations individuals were in moderate/average body condition [60, 79]. Despite the range in body conditions observed (Fig 3), most collars were observed fitting correctly (94% of observations for both horses and burros). Snug collar fit was reported 24 times in burros with a mean body condition score of 4.8 (Fig 3) and on 2 horses, one of which had a body condition score of 6 (moderately fleshy) and the other had no body condition reported. There was no difference in body condition score between burros reported with snug collars and those with correctly fitting collars (*X*^2^ = 1.1568, df = 6, *P* = 0.979). We had 105 observations of loose fit on burros (99 with body condition reported) and 222 observations of horses (216 with body condition reported), tending to be observed on individuals in lower body condition (burros: X^2^ = 13.074, df = 6, *P* = 0.042; horses: X^2^ = 36.874, df = 4, *P* = <0.0001; Fig 3).

Spun collars (i.e., upside down on the animal’s neck) were reported on 6% of burro observations and 2% of horse observations (Table 3) and were not associated with reports of collars being loose on either species; spun collars were reported in only 10% of loose observations for burros, and 4% for horses. In burros, spun collars were associated with superficial effects seen roughly half the time (superficial effects seen in 63 of 127 spun collar observations [49%]), whereas for horses, superficial effects were rarely seen associated with a spun collar (8 out of 65 observations with a spun horse collar [12%]).

**Fig 4:**
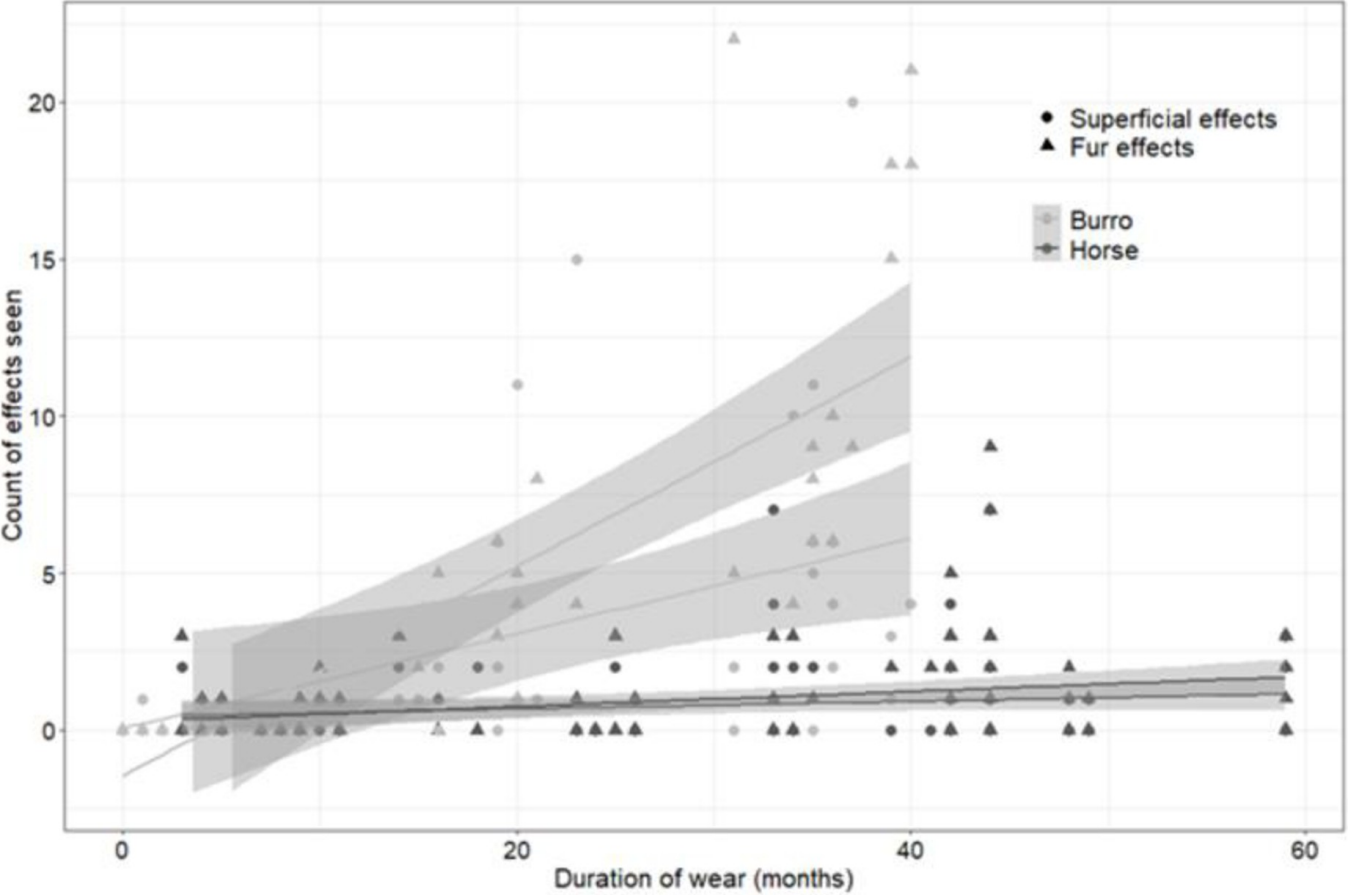
The relationship between duration of wearing a collar and number of effects observed on horses and burros in the western United States, 2016–2020. Regression lines for horses are shown in black, with lines for burros shown in grey, along with data points (triangles represent effects to fur and dots denote superficial effects), with shaded 95% confidence intervals also shown. The steeper slope for burros is a reflection of increased fur effects (indented fur, broken fur, sweaty neck) over time for burros with longer duration of wear. Superficial effects (chafe, wound, scab) also increased for burros with longer duration of wearing a collar, but the slope was lower than for fur effects. Horses had slight increases in fur effects over longer duration of wear but no increases in superficial effects.

Superficial effects were reported in 11% (n = 256) of 2,258 burro observations and fur effects in 25% (n = 554). Horses had superficial effects in 2% (n = 79) of 4,307 observations and fur effects in 3% (n = 107). respectively of 4,307 observations. There was an effect of time wearing a collar on the cumulative sum of fur effects for both burros and horses (burros: r_s_ = 0.87, P = <0.0001; horses: r_s_ = 0.31, P = 0.002; Fig 4), but there was only an effect of time wearing a collar on superficial effects for burros (r_s_ = 0.62, P = <0.0001) with no effect on horses (r_s_ = 0.11, P = 0.2862). In our assessment of the potential for progression of effects, we found 10% of effects displayed a progression (*n* = 48 observations). However, the majority of observations did not develop into serious effects. Eighty-six percent of observations that started with a chafe stayed as a chafe (*n* = 206), 79% of incidents starting with indented fur stayed as indented fur (*n* = 134), 63% of incidents starting with a sweaty neck stayed as a sweaty neck (*n* = 5) until the condition was no longer observed on the neck because it resolved.

Collars sometimes moved into a wrong position, shifting forward over the ears. We observed this condition on 19 horses across study areas (Adobe Town: 8; Conger: 5; Frisco: 6), and 1 burro at Lake Pleasant. Most collars went over the ears in summer (*n* = 12), although it occurred in all seasons (spring: 2; fall: 2; winter: 4). For horses, 79% of occurrences of collars going over the ears happened within the first year after deployment, with the remaining 21% in the second year. By the third study year there were no further observations of collars going forward over the ears. On five occasions, collars were observed in the wrong position but subsequently righted themselves on the neck.

After collar removal, we examined the necks of 16 horses from Frisco who had been wearing collars from 41 to 44 months (mean = 43.06 months ± 1.12 SD). Half of these horses (*n* = 8) had minor physical effects: indented fur, chafing, or small scabs; the other 8 had no observable physical effects. At Conger we examined the necks of 28 horses who had been wearing collars from 33 to 59 months (mean = 45.39 months ± 11.16 SD). We manually removed 12 collars from horses and the remaining 16 had their collars dropped remotely 11 months prior to being examined. All 12 of the Conger horses whose collars were removed manually had evidence of some minor chafing and 8 had indented fur. Three of the 16 horses who had not been wearing a collar for almost a year had small marks on their necks which were possibly related to wearing a collar (chafe marks: *n* = 2, indented fur: *n* = 1). Seven horses at Conger and 1 at Frisco who had been wearing collars for 44 to 59 months developed small callouses on their neck (~2 cm diameter), some of which were fluid filled, under the area that would have been covered by the collar. These did not require veterinary treatment, were easily palpated, and horses did not react to palpation. We found no scarring or leukotrichia (patches of white hair related to skin trauma) on any horse.

**Fig 5:**
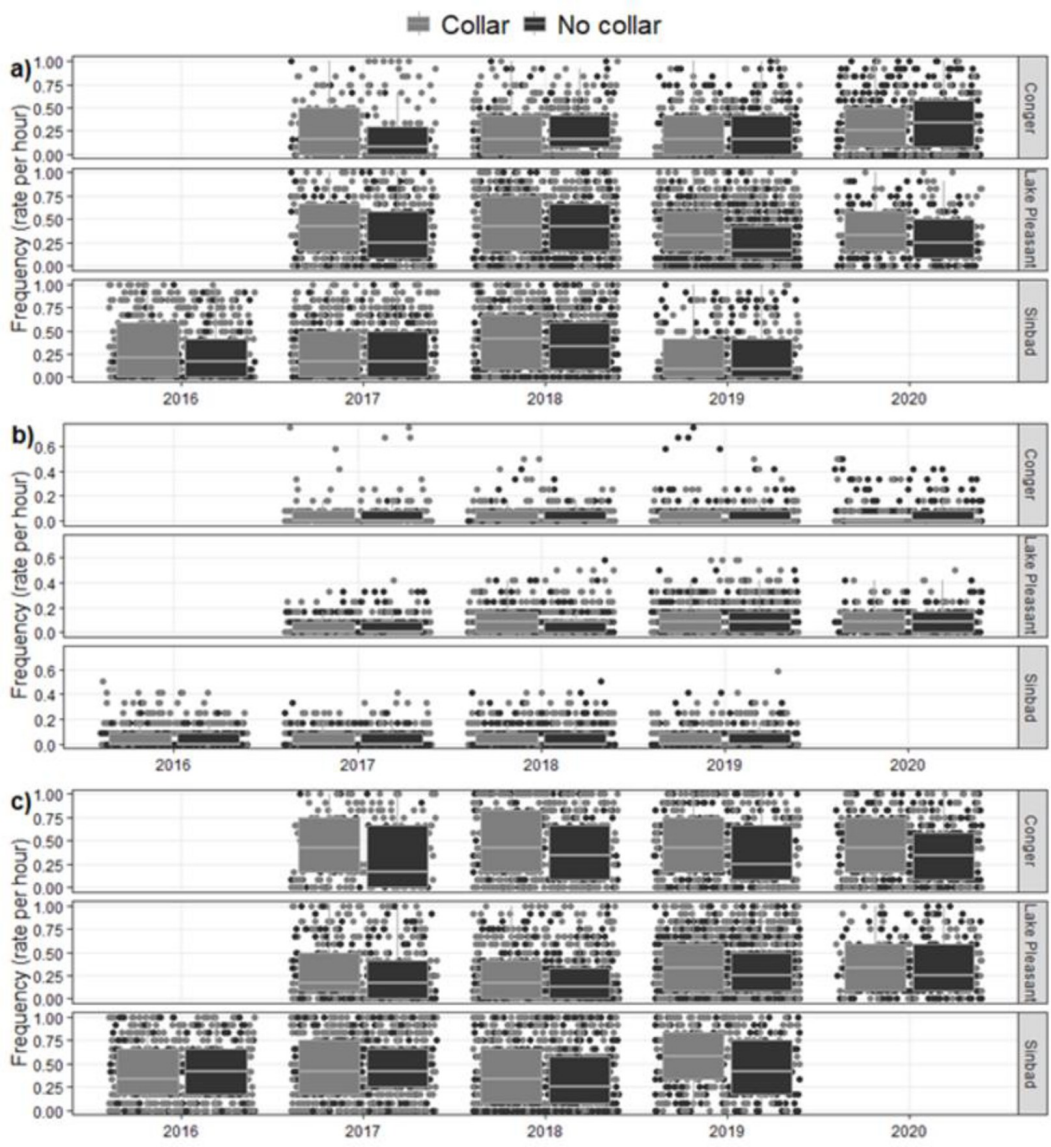
Frequency of a) feeding, b) moving, and c) standing behaviors between March and September 2016–2020, by burros at Sinbad Herd Management Area (HMA), Lake Pleasant HMA, and horses at Conger HMA, Utah, USA, comparing collared and uncollared individuals. Box plots show median (horizontal line) and interquartile ranges, and all data points are shown jittered to show detailed spread of points.

There was little effect of collars on maintenance behaviors. For Sinbad burros, the best models explaining feeding, moving, and standing were year or collar + year (Fig 5 and S2 Table), with no inference from the presence of collars for any behavior (feeding: Wald 95% confidence intervals (CI): -0.215–0.108; moving: 95% CI: -0.414–0.297, standing: 95% CI -0.205–0.056). At Lake Pleasant collar + year was the best model for all behaviors. Collared burros there tended to spend more time feeding (95% CI: -0.354 - -0.090; Fig 5 and S3 Table) and standing (95% CI: -0.285 - -0.009), but there was no effect on time moving (95% CI:-0.299–0.181). There was no effect of wearing a collar on feeding by horses at Conger (95% CI: -0.114–0.273; Fig 5 and S4 Table), and no effect on moving even though collar was the top model (95% CI: -0.326–0.556), but there was some effect on standing, with collared animals tending to stand more (95% CI: -0.324– -0.035).

There were no adult mortalities related to telemetry collars. There was no difference in annual survival of foals born to collared or uncollared horse mares (collared mares: mean 95.6% ± 4.3; uncollared mares: mean 94.9% ± 4.9; Student’s t = -0.08, df = 3, P = 0.9381), or collared or uncollared burro jennies (collared jennies: mean 86.7% ± 13.7; uncollared jennies: mean 79.2% ± 14.7; t = 0.38, df = 4, P = 0.7214).

We made 160 attempts to remotely drop collars from 121 horses and burros (Table 4 and S2 Fig), with some animals receiving multiple attempts in succession, or on different days. Most of these attempts (*n* = 129) were made using a handheld device provided by the collar vendor to remotely drop the collar using an ultra-high frequency (UHF) radio signal; these devices had a 53% success rate (*n* = 68) at removing the collar. We made another 19 attempts to drop Iridium collars using the 2-way Iridium communication through a web service, which had a 42% success rate (*n* = 8). No attempt to drop was made for 2 collars at Frisco, 4 at Lake Pleasant, and 14 at Sinbad because collars were no longer transmitting/communicating with satellites. The collar removals necessary during our 4-year study period were for collars that stopped communicating with the web service (technology failures) or collars that went over the ears into an incorrect position on the neck, which had potential to cause discomfort or wounding and so were attempted to be removed at first observation.

**Table 4:** Number of remote collar removal attempts at each study site (using handheld ultra-high radio signal transmitters, Iridium web service, and timed-release). Attempts resulted in either a failed attempt, where the collar failed to release after the drop command was sent, or a successful drop where the collar came off the animal. Number of failed and successful attempts are shown, with percentage of all attempts made at that site in parentheses. Note that repeat attempts were made to drop some horse collars. Collars that were not dropped successfully were removed when individuals came in during the gather at the end of the study.

Species	HMA	No. collars attempted	No. attempts	Successful drops	Unsuccessful drops
**Horse**	Adobe Town	38*	58	35 (60%)	23 (40%)
**Horse**	Conger	27	43	14 (33%)	29 (67%)
**Horse**	Frisco	26	28	16 (57%)	12 (43%)
**Burro**	Lake Pleasant	23	23	17 (74%)	6 (26%)
**Burro**	Sinbad	8	8	6 (75%)	2 (25%)

*One individual was bait-trapped during the study when her collar went over the ears and the remote drop did not function. This individual was bait-trapped, her collar re-fit and a new remote drop device attached.

In addition to the remote drop off, all collars were equipped with a timed-release drop off scheduled to deploy at the end of the study. At Adobe Town 13 collars were on horses at the time of the scheduled automatic release (24 months), and 12 dropped off as expected. At the other 4 study sites the number of weeks scheduled until timed release drops exceeded the study period, so the success of timed release drop off devices could not be evaluated.

There was no correlation between time since deployment of the collar and non-response of the collar drop-off mechanism (r_s_ = 0.254, P = 0.082; S2 Fig). There was a tendency within the first two years after collar deployment for attempts to result in successful drops (80% successful, 20% failed; *n* = 65) with reduced success in years 3 and 4 (38% successful, 62% failed; *n* = 95) but there was no difference in success rate among years (Kruskal-Wallis H = 2.185, df = 3, P = 0.5349).

## Discussion

Any animal that is part of a research study should be treated humanely [1, 2] and not undergo injury from the study [43]. Further, it is increasingly mandated that studies adhere to approved oversight and protocols that minimize stress, pain, and injury [3]. This is important not only for ethics within the wildlife profession and transparency with the public [4, 5], but for the research itself, because injuries can influence animal behavior that may affect study findings [73]. Marking and telemetry tracking devices should not influence study animals [74]. If collars do not fit properly or cause discomfort, it can bias research results [75]. In our study, we saw no observable physical effects for the majority (85%) of observations of horses and burros wearing telemetry collars, and of those effects observed the majority were inconsequential effects to fur (sweaty neck, indented fur, broken fur) with some superficial effects to skin surface (chafe, wound, scab) that all healed within 4–6 weeks, were less than 2.4 cm in diameter, and did not grow larger or develop into more serious conditions. We did not need to remove a collar from a study animal for any of these effects because they never became acute. The only collar removals necessary during our 4-year study period were for collars that stopped communicating with the web service or went over the ears into an incorrect position. We had several collars move into wrong position and then into correct position on their own, but this was not common. We relied on the web service drop-off command or the remote release mechanism on each collar to remove collars when needed. The BLM required that we include a remote release mechanism as well as a timed release drop off on each collar, and we found these remote capabilities to be important components for telemetry collars on free-roaming equids. In cases where collars did not drop off on command, which was mostly in years 3 and 4 of our study, contingency plans had been established in management plans (bait trap, corral, or manual capture) to assure removal and retrieval of the collar.

Relative to collars going over the ears, there appeared to be an acclimation period by horses to wearing telemetry collars, in which most incidents of over-the-ears occurred in the first year of collar wear, with fewer in year 2, and none in years 3 and 4. In addition, collars that were deployed in winter (e.g., Adobe Town HMA) more frequently went over ears than those deployed in summer or fall. In northern latitudes where horse pelage grows very thick, collars may have been deployed too loosely in an effort to allow space for summer weight gain. However, the assumption of increased weight gain leading to slightly greater neck thickness in summer is potentially offset by decreased summer fur thickness in these populations.

The single incident in which a burro collar went over the ears was an anomaly and somewhat suspicious. Burros have a larger face-to-neck ratio than horses, and their relatively large ears make it difficult for a collar to move over the ears into the wrong position. The one case of this occurring took place in an area of Lake Pleasant HMA with high human visitation (near a boat ramp) where burros were acclimated to humans, and humans approach them and feed them. We suspect someone grabbed ahold of the collar and when the burro pulled away, it was forced over the ears. It did not occur on any Sinbad burros where there was low human presence.

We tried to assess whether seemingly inconsequential effects (sweaty neck, indented fur) might lead to potentially consequential effects (wound, scab) and found little evidence for any further progression nor any pattern of progression. It could be that our data lacked the temporal scale needed to assess a linear progression of effects; we used 10-day observation sessions, followed by 4 days off, then another 10-day session. On this schedule, most effects were reported only once and subsequently resolved, or stayed the same over one or several observation periods and did not progress to anything more severe. Furthermore, we also did not find escalating effects over time in our regression analysis for horses; effects to fur (sweaty neck, indented fur, broken fur) were the only physical effects that cumulatively (and only slightly) increased over time for horses. Burros appeared to be more sensitive to duration of collar wear than horses, exhibiting increases in fur effects like horses, but also increases in superficial effects over time. We are unsure why some burros appear to be more sensitive but suggest different belting material should be tested on burros to make collars more supple. Burro collars that are soft and have rounded wide edges are less likely to cause superficial effects from rubbing. Further, making sure collars are fit snugly so they do not move up and down the neck is important for both burros and horses to reduce potential effects from rubbing such as chafing, although our experience was that burro collars should not be fit as snugly as horses. Additionally, we suggest testing more breathable, supple fabrics for collar belting that could ameliorate neck sweat and broken fur we observed. Collars made with heavy durable nylon or canvas-type fabric have worked well on other species, such as Asian elephants (*Elaphus maximus*) [76], and this material may be appropriate for equids as well. Testing different belting material was outside the scope of this study but is an area for future investigation. Another consideration is collar weight. We applied collars that were appropriate weight for the body size of horses and burros [77, 78], but testing different collar weights, such as lighter collars, would also be useful to assess how it impacts minor collar effects.

Important parameters in the model that determined if fur or superficial effects were observed included study area and distance to animal. In some HMAs, individuals were easier to approach, so the closer the observer was to the collared individual, the better opportunity there was to scrutinize the neck. Lake Pleasant and Sinbad HMAs were more conducive to closer observation distances, whereas in Adobe Town, Conger and Frisco HMAs study individuals were less approachable, and observations from greater physical distance required binoculars and spotting scopes. Although the model indicated that burros are more likely to be observed with collar effects, intuitively it seems likely that burros were reported to have continuous minor physical effects from collars because they were observed from closer distances. Our best model was not strong and only explained 25% of the variance, most likely because we had such a low number of any physical effects being observed at all (low sample size of effects). Conditions that were easy to view regardless of distance to animal included spun collars, and collars in wrong position over the ears. Both of those conditions were more visible and obvious and could be identified from close or far distances to the collared individual.

Body condition, a qualitative estimate of subcutaneous adipose tissue storage correlated with body mass [79, 80], was not a good predictor of whether a collar was reported as too loose or too snug, primarily because most individuals maintained consistent and ideal body condition (i.e., scores between 4 and 6) throughout the 4 years of the study. Collars in our study remained symmetrical with correct tightness on the neck in the majority of horse and burro observations (94%) but in a few cases where the collar was reported as “loose’ on horses, we had several observations of physical effects that may have been caused by rubbing. Loose collars presumably permit more collar movement on the neck and therefore the potential for rubbing, which could lead to fur or superficial effects, although we did not find a clear pattern in our dataset. Physical effects that may have been related to collars being too loose or too snug were only reported on a small proportion of observations suggesting a collar appearing too loose or too snug may not be a useful indicator of effects. However, collars should be placed flush and snug on the neck of horses just behind the ears (S1–S9 Photos) to reduce the potential for friction. Changes in neck size and seasonal weight gain and loss have been reported to affect collar fit [81, 82], but we did not find evidence for any of these conditions in our study. We maintain that the presence of a remote drop-off mechanism and regular observations of collared individuals can alleviate any conditions that arise.

Our behavior results indicated minimal effects on horse or burro maintenance behaviors; results for horses showed they stood slightly more than uncollared horses, and collared burros were found to forage and stand more at Lake Pleasant; no behavioral effects of wearing collars were seen on burros at Sinbad. Changes in movement have been reported in an African equid: collared plains zebras had lower movement rates than uncollared [44], but in our study it is likely that factors such as resource availability and time of year were stronger predictors of animal movement and behavior than wearing a collar [20]. Any minor differences in observed behavior were not found to be correlated with any negative demographic effect.

In our study, we did not equip male equids with radio collars, primarily due to sex-specific behaviors that damage collars and have been reported in other studies [83–86]. Zebra stallions were not tracked in one study due to the risk of damage to collars during fighting [44]. Similarly, we did not collar burro jacks or horse stallions due to challenges noted in previous studies with aggressive fighting that damaged collars [87] or collar fit problems [50, 58].

There were no mortalities related to collars in any of the 5 populations we studied and survival of foals born to collared and uncollared mares and jennies did not differ. Coupled with the low frequency of meaningful physical effects and the lack of any severe wounding, all these findings suggest the biological consequences of collars on free-roaming equid welfare and survival is insignificant, although frequently monitoring individually collared equids is warranted and necessary. Based on our remote collar drop off results, contingency plans should be established when collars are deployed to enable removing collars manually if needed. We conclude that with regular monitoring, collaring of free-roaming equids poses minimal risks to animal welfare and is a safe and essential tool to increase our understanding of equid spatial ecology, habitat use, and interactions with other wildlife.

## Supporting information

S1 Table Candidate models, number of parameters (K), ΔAIC_c_, Akaike weight (w_i_), and log-likelihood (LL) for evaluating the role of study area (Adobe Town, Conger, Frisco, Lake pleasant, and sinbad herd management areas), species (horse or burro), distance of the observer from theanimals, observation quality code (obs code), body condition (BC), year, season, and which side of the animal’s neck was seen (one side, both sides, no sides) for individual physical effects observed among feral horses and burros in the western United States between 2016 and 2020. Physical effects measured were: a) sweaty neck, b) indented fur, c) broken fur, d) chafe, e) scab, f) wound, or g) over the ears.
(PDF)

S2 Table Candidate models, number of parameters (K), ΔAIC_c_, Akaike weight (w_i_), and log-likelihood (LL) for evaluating the role of study year (2016 to 2019, inclusive) and wearing a collar on maintenance behaviors (a) feeding; b) moving; c) standing) of burros at Sinbad Herd Management Area, Utah, USA.
(PDF)

S3 Table Candidate models, number of parameters (K), ΔAIC_c_, Akaike weight (w_i_), and log-likelihood (LL) for evaluating the role of study year (2017 to 2020, inclusive) and wearing a collar on maintenance behaviors (a) feeding; b) moving; c) standing) of burros at Lake Pleasant Herd Management Area, Arizona, USA.
(PDF)

S4 Table Candidate models, number of parameters (K), ΔAIC_c_, Akaike weight (w_i_), and log-likelihood (LL) for evaluating the role of study year (2017 to 2020, inclusive) and wearing a collar on maintenance behaviors (a) feeding; b) moving; c) standing) of horses at Conger Herd Management Area, Utah, USA.
(PDF)

S5 Table Length of time horses and burros were held in a chute to affix collars and apply freeze marks, compared to animals that were not collared and only received a freeze mark, for a study of potential effects of collars on free-roaming equids, 2016–2020. Data are from 4 populations in the western United States: burros in Lake Pleasant and Sinbad Herd Management Areas, and horses in Conger and Frisco Herd Management Areas. Numbers shown indicate mean ± standard deviation in minutes, with minimum and maximum time held shown in parentheses.
(PDF)

S1 Fig Mean estimated distance (± standard deviation) from which equids were observed (horses at Adobe Town, Conger, and frisco herd management areas and burros at Lake Pleasant and sinbad herd management areas) and observation quality code assigned by observers. Observation quality score was a qualitative measure, ranked from 1 (lowest, worst view) to 5 (highest, best view) and included an assessment of how well the animal was viewed and whether both sides of the neck were observed. As distance to individual being observed decreased, observation quality code increased.
(PDF)

S2 Fig Attempts to remotely release collars on horses at Adobe Town, Conger, and frisco herd management areas (HMAs) and burros at Lake pleasant and sinbad HMAs using handheld remote drop devices or the Iridium web service, relative to time since collar deployment. Attempts resulted in either a successful drop where the collar came off the animal, or a failed attempt where the collar failed to release after the drop command was sent. We made attempts to drop collars remotely at the end of the study primarily and not at different times throughout the study. Thus, we are unsure if collar drop offs may have failed sooner than at the time we tried them.
(PDF)

S1 Text Description of technique used to affix GPS collars to the necks of free-roaming equids in a study of potential effects of collars on free-roaming equids in five populations in the western United States, 2016–2020. (PDF)

S1 Photo Image of two collared adult horses in a holding corral, demonstrating correct position with battery on the bottom and GPS unit on the top of neck, for a study of potential effects of collars on free-roaming equids, 2016–2020, Wyoming, USA. Photo credit K.A. Schoenecker.
(PDF)

S2 Photo Image of GPS collaring of horses by accessing the horse’s neck through a side panel of the padded hydraulic squeeze chute, for a study of potential effects of collars on free-roaming equids, 2016–2020, USA. The individuals in this photograph have given written informed consent to publish this photo.
(PDF)

S3 Photo Placing the collar on the neck of a horse while a plastic bin lid is used as a guard, held along the mare’s head facing forward to dissuade the mare from swinging her head around to bite. This visual block can also aid in keeping the mare calm, similar to the effect of blindfolds that are used for ungulate live captures. This collaring application technique was developed for a study of potential effects of collars on free-roaming equids, 2016–2020, USA. The individuals in this photograph have given written informed consent to publish this photo.
(PDF)

S4 Photo Placing bolts on the collar strap before moving the collar up the neck into correct position behind the ears and then tightening the collar, for a study of potential effects of collars on free-roaming equids, 2016–2020, USA. The black rectangle on the side of the collar is the remotely triggerable drop off mechanism. The individuals in this photograph have given written informed consent to publish this photo.
(PDF)

S5 Photo Burro standing with minimal restraint in a transportable fly chute in the Lake Pleasant Herd Management Area, Arizona, USA, for a study of potential effects of collars on free-roaming equids, 2016–2020. Photo credit: K.A. Schoenecker.
(PDF)

S6 Photo Image of one collared burro and one uncollared burro grazing at the Sinbad Herd Management Area, Utah, USA in a study of potential effects of collars on free-roaming equids, 2016–2020. Photo credit: M.J. Cole.
(PDF)

S7 Photo Image of horse group with two collared mares and several uncollared mares grazing at the Conger Herd Management Area, Utah, USA, for a study of potential effects of collars on free-roaming equids, 2016–2020. Photo credit: S.R.B. King.
(PDF)

S8 Photo Image of two collared horses grazing at the Frisco Herd Management Area, Utah, USA, for a study of potential effects of collars on free-roaming equids, 2016–2020. Photo credit: R. Fawbush.
(PDF)

S9 Photo Close up image of a collared mare grazing. A remotely triggerable drop off mechanism is visible on the side of the collar. Photo credit: S.R.B. King.
(PDF)
