# Supplementary material for: Effects of telemetry collars on two free-roaming feral equid species
Source: PLoS One. 2024 May 30;19(5):e0303312. doi: 10.1371/journal.pone.0303312 (PMC11139308; doi:10.1371/journal.pone.0303312)
Supplement: S1 Table — Physical effects measured were: a) sweaty neck, b) indented fur, c) broken fur, d) chafe, e) scab, f) wound, or g) over the ears. (PDF) [file pone.0303312.s001.pdf]

a) Sweaty neck

Model	AICc	K	ΔAIC_c	w_i	LL
Study area + log10(distance)	332.24	6	0	0.46	-160.11
Study area + season	332.37	8	0.13	0.43	-158.17
Study area + obs code	337.68	9	5.44	0.03	-159.82
Study area + one side	338.99	6	6.75	0.02	-163.49
Study area	339	5	6.76	0.02	-164.5
Study area + both sides	339.84	6	7.61	0.01	-163.91
Study area + no sides	340.47	6	8.23	0.01	-164.23
Species	340.95	2	8.71	0.01	-168.47
Study area + year of study	340.99	6	8.75	0.01	-164.49
Log10(distance)	348.47	2	16.23	0	-172.24
Study area + BC	348.82	12	16.58	0	-162.38
Obs code	354.59	5	22.35	0	-172.29
Season	369.97	4	37.73	0	-180.98
Intercept	376.47	1	44.24	0	-187.24
Year of study	376.57	2	44.33	0	-186.28

b) Indented fur

Model	AICc	K	ΔAIC_c	w_i	LL
Study area + obs code	2138.96	9	0	1	-1060.47
Study area + log10(distance)	2206.37	6	67.41	0	-1097.18

This draft manuscript is distributed solely for the purposes of review. Its content is deliberative and pre-decisional, so it must not be disclosed or released by reviewers. Because this manuscript has not been approved by the U.S. Geological Survey (USGS), it does not represent any official USGS finding or policy.

Study area + season	2234.95	8	95.99	0	-1109.46
Study area + year of study	2244.52	6	105.56	0	-1116.25
Study area + one side	2245.17	6	106.21	0	-1116.58
Study area + both sides	2248.32	6	109.36	0	-1118.15
Study area	2267.16	5	128.2	0	-1128.58
Study area + BC	2267.24	12	128.27	0	-1121.59
Study area + no sides	2268.89	6	129.93	0	-1128.44
Species	2325.98	2	187.02	0	-1160.99
Obs code	2412.75	5	273.78	0	-1201.37
Log10(distance)	2529.44	2	390.47	0	-1262.72
Season	2751.65	4	612.69	0	-1371.82
Intercept	2786.44	1	647.48	0	-1392.22
Year of study	2788.32	2	649.35	0	-1392.16

c) Broken fur

Model	AICc	K	ΔAIC_c	w_i	LL
Study area + obs code	1616.15	9	0	1	-799.06
Study area + log10(distance)	1627.23	6	11.08	0	-807.61
Study area + both sides	1644.44	6	28.29	0	-816.21
Study area + one side	1651.04	6	34.89	0	-819.51
Study area + year of study	1664.47	6	48.32	0	-826.23
Study area + season	1665.57	8	49.42	0	-824.77

Study area + no sides	1678.66	6	62.51	0	-833.32
Study area	1683.98	5	67.83	0	-836.98
Study area + BC	1693.1	12	76.95	0	-834.52
Species	1700.3	2	84.15	0	-848.15
Obs code	1736.39	5	120.25	0	-863.19
Log10(distance)	1743.44	2	127.29	0	-869.72
Season	1928.21	4	312.06	0	-960.1
Intercept	1946.73	1	330.58	0	-972.36
Year of study	1948.73	2	332.58	0	-972.36

d) Chafe

Model	AICc	K	ΔAIC_c	w_i	LL
Study area + year of study	1663.41	6	0	0.94	-825.7
Study area + obs code	1668.88	9	5.47	0.06	-825.42
Study area + log10(distance)	1702.61	6	39.2	0	-845.3
Study area + one side	1722.02	6	58.61	0	-855
Study area + both sides	1725.44	6	62.03	0	-856.71
Study area + season	1741.87	8	78.47	0	-862.92
Study area + BC	1743.79	12	80.38	0	-859.87
Study area	1752.58	5	89.17	0	-871.28
Study area + no sides	1754.49	6	91.08	0	-871.24
Species	1826.68	2	163.28	0	-911.34

Log10(distance)	1839.67	2	176.26	0	-917.83
Obs code	1865.65	5	202.24	0	-927.82
Year of study	2050.57	2	387.16	0	-1023.28
Season	2058.72	4	395.31	0	-1025.36
Intercept	2064.93	1	401.53	0	-1031.47

e) Scab

Model	AICc	K	ΔAIC_c	w_i	LL
Study area + obs code	506.93	9	0	1	-244.45
Study area + log10(distance)	527.61	6	20.68	0	-257.8
Obs code	527.93	5	21	0	-258.96
Study area + season	528.77	8	21.84	0	-256.37
Study area + year of study	529.85	6	22.92	0	-258.92
Study area + one side	531.95	6	25.02	0	-259.97
Study area + no sides	532.72	6	25.79	0	-260.35
Study area	533.97	5	27.03	0	-261.98
Study area + both sides	534.67	6	27.74	0	-261.33
Species	537.69	2	30.76	0	-266.85
Study area + BC	538.72	12	31.79	0	-257.33
Log10(distance)	540.54	2	33.61	0	-268.27
Year of study	564.98	2	58.04	0	-280.49
Season	571.79	4	64.85	0	-281.89

Intercept	574.86	1	67.93	0	-286.43

f) Wound

Model	AICc	K	ΔAIC$_c$	w$_i$	LL
Study area + obs code	280.99	9	0	0.77	-131.48
Study area + log10(distance)	283.49	6	2.5	0.22	-135.74
Study area + one side	290.56	6	9.57	0.01	-139.27
Obs code	291.17	5	10.18	0	-140.58
Study area + year of study	293.06	6	12.07	0	-140.52
Study area + both sides	293.46	6	12.47	0	-140.72
Study area	297.05	5	16.06	0	-143.52
Study area + no sides	297.92	6	16.93	0	-142.95
Study area + season	300.34	8	19.36	0	-142.16
Year of study	306.35	2	25.36	0	-151.18
Study area + BC	309.61	12	28.62	0	-142.78
Log10(distance)	309.9	2	28.91	0	-152.95
Intercept	323.2	1	42.21	0	-160.6
Species	324.66	2	43.67	0	-160.33
Season	327.76	4	46.77	0	-159.88

g) Over the ears

Model	AICc	K	ΔAIC$_c$	w$_i$	LL

Study area + log10(distance)	290.69	6	0	0.34	-139.34
Study area + obs code	291.13	9	0.45	0.27	-136.55
Study area + year of study	291.9	6	1.22	0.19	-139.94
Study area + one side	293.03	6	2.34	0.11	-140.51
Study area + both sides	294.6	6	3.91	0.05	-141.29
Study area + season	295.58	8	4.9	0.03	-139.78
Study area	298.66	5	7.97	0.01	-144.32
Study area + no sides	300.24	6	9.56	0	-144.11
Species + study area	300.66	6	9.98	0	-144.32
Study area + BC	301.23	12	10.55	0	-138.59
Species	312.13	2	21.44	0	-154.06
Year of study	313.87	2	23.18	0	-154.93
Obs code	317.64	5	26.95	0	-153.81
Season	319.07	4	28.38	0	-155.53
Intercept	323.2	1	32.51	0	-160.6
Log10(distance)	323.53	2	32.84	0	-159.76
