# Supplementary material for: Effects of telemetry collars on two free-roaming feral equid species
Source: PLoS One. 2024 May 30;19(5):e0303312. doi: 10.1371/journal.pone.0303312 (PMC11139308; doi:10.1371/journal.pone.0303312)
Supplement: S2 Table — Candidate models, number of parameters (K), ΔAICc, Akaike weight (wi), and log-likelihood (LL) for evaluating the role of study year (2016 to 2019, inclusive) and wearing a collar on maintenance behaviors (a) feeding; b) moving; c) standing) of burros at Sinbad Herd Management Area, Utah, USA. (PDF) [file pone.0303312.s002.pdf]

a) Feeding

Model	AICc	K	ΔAIC_c	w_i	LL
Year	2224.84	7	0	0.65	-1105.39
Collar + year	2226.19	8	1.35	0.33	-1105.06
Collar * year	2231.63	11	6.79	0.02	-1104.75
Intercept only	2272.75	4	47.91	0	-1132.36
Collar	2274.46	5	49.62	0	-1132.22

b) Moving

Model	AICc	K	ΔAIC_c	w_i	LL
Year	790.84	5	0	0.36	-390.41
Collar + year	790.92	7	0.08	0.35	-388.43
Collar * year	792.14	6	1.3	0.19	-390.05
Intercept only	793.58	8	2.74	0.09	-388.75
Collar	800.62	12	9.78	0	-388.24

c) Standing

Model	AICc	K	ΔAIC_c	w_i	LL
Year	3542.97	5	0	0.66	-1766.47
Collar + year	3544.94	6	1.98	0.25	-1766.46
Collar * year	3546.84	9	3.87	0.1	-1764.39
Intercept only	3558.77	2	15.81	0	-1777.38

Collar	3560.66	3	17.69	0	-1777.33
