# Supplementary material for: Effects of telemetry collars on two free-roaming feral equid species
Source: PLoS One. 2024 May 30;19(5):e0303312. doi: 10.1371/journal.pone.0303312 (PMC11139308; doi:10.1371/journal.pone.0303312)
Supplement: S3 Table — Candidate models, number of parameters (K), ΔAICc, Akaike weight (wi), and log-likelihood (LL) for evaluating the role of study year (2017 to 2020, inclusive) and wearing a collar on maintenance behaviors (a) feeding; b) moving; c) standing) of burros at Lake Pleasant Herd Management Area, Arizona, USA. (PDF) [file pone.0303312.s003.pdf]

a) Feeding

Model	AICc	K	ΔAIC_c	w_i	LL
Collar + year	1976.59	6	0	1	-982.27
Collar	1990.61	3	14.02	1	-992.3
Year	1996.79	5	20.2	1	-993.38
Intercept Only	2007.78	2	31.2	1	-1001.89
Collar * Year	Insufficient information to fit model				

b) Moving

Model	AICc	K	ΔAIC_c	w_i	LL
Collar + year	797.35	7	0	0.76	-391.64
Year	800.14	8	2.79	0.19	-392.02
Intercept Only	803.69	5	6.33	0.03	-396.82
Collar	805.06	6	7.71	0.02	-396.5
Collar * year	807.17	12	9.81	0.01	-391.48

c) Standing

Model	AICc	K	ΔAIC_c	w_i	LL
Year	1518.68	5	0	0.65	-754.32
Collar + year	1520.65	6	1.97	0.24	-754.3
Collar * year	1523.01	9	4.33	0.07	-752.45
Intercept Only	1525.4	2	6.72	0.02	-760.7

Collar	1527.26	3	8.59	0.01	-760.62
