# Supplementary material for: Effects of telemetry collars on two free-roaming feral equid species
Source: PLoS One. 2024 May 30;19(5):e0303312. doi: 10.1371/journal.pone.0303312 (PMC11139308; doi:10.1371/journal.pone.0303312)
Supplement: S4 Table — Candidate models, number of parameters (K), ΔAICc, Akaike weight (wi), and log-likelihood (LL) for evaluating the role of study year (2017 to 2020, inclusive) and wearing a collar on maintenance behaviors (a) feeding; b) moving; c) standing) of horses at Conger Herd Management Area, Utah, USA. (PDF) [file pone.0303312.s004.pdf]

a) Feeding

Model	AICc	K	ΔAIC_c	w_i	LL
Collar	746.69	3	0	0.25	-370.33
Intercept Only	746.91	2	0.22	0.23	-371.45
Collar + year	746.93	6	0.24	0.23	-367.41
Year	747.64	5	0.95	0.16	-368.78
Collar * year	747.98	9	1.29	0.13	-364.87

b) Moving

Model	AICc	K	ΔAIC_c	w_i	LL
Year	42.6	5	0	0.43	-16.26
Intercept Only	43.31	2	0.71	0.73	-19.65
Collar + year	44.58	6	1.97	0.89	-16.23
Collar	45.3	3	2.7	1	-19.64
Collar * Year	Insufficient information to fit model				

c) Standing

Model	AICc	K	ΔAIC_c	w_i	LL
Intercept Only	1004.44	2	0	0.45	-500.21
Collar	1004.49	3	0.05	0.44	-499.23
Collar + year	1008.5	6	4.06	0.06	-498.19

Year	1008.63	5	4.19	0.06	-499.27
Collar * year	1013.71	9	9.27	0	-497.74
