# Supplementary material for: Effects of telemetry collars on two free-roaming feral equid species
Source: PLoS One. 2024 May 30;19(5):e0303312. doi: 10.1371/journal.pone.0303312 (PMC11139308; doi:10.1371/journal.pone.0303312)
Supplement: S5 Table — Data are from 4 populations in the western United States: burros in Lake Pleasant and Sinbad Herd Management Areas, and horses in Conger and Frisco Herd Management Areas. Numbers shown indicate mean ± standard deviation in minutes, with minimum and maximum time held shown in parentheses. (PDF) [file pone.0303312.s005.pdf]

Species	Collared (min-max)	Non-collared (min-max)	Mean added time for collaring
**Burro**	10.9 ± 5.5 (4–32)	6.3 ± 4.5 (1–26)	4.6 ± 1.0
**Horse**	8.1 ± 2.8 (4–16)	3.6 ± 1.6 (2–9)	4.5 ± 1.2
