# Supplementary figures and images for: Effects of telemetry collars on two free-roaming feral equid species
Source: PLoS One. 2024 May 30;19(5):e0303312. doi: 10.1371/journal.pone.0303312 (PMC11139308; doi:10.1371/journal.pone.0303312)

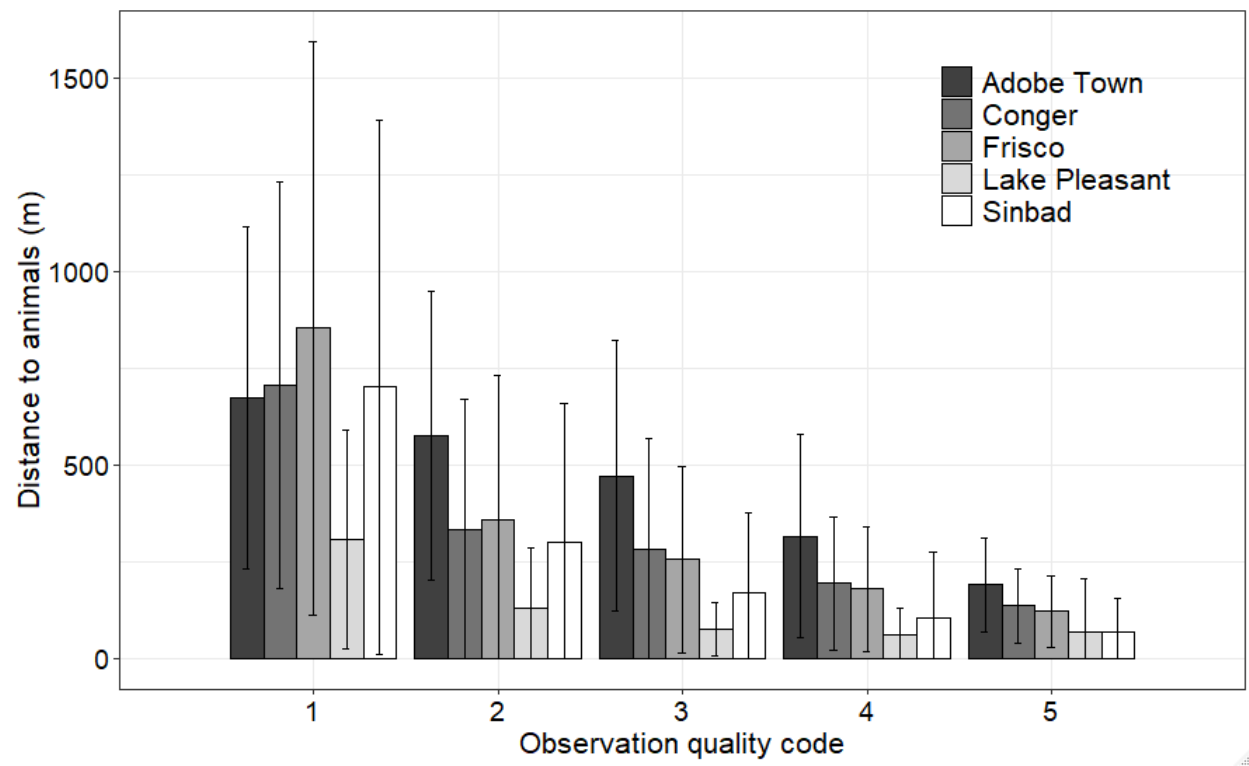

Supplement: S1 Fig — Observation quality score was a qualitative measure, ranked from 1 (lowest, worst view) to 5 (highest, best view) and included an assessment of how well the animal was viewed and whether both sides of the neck were observed. As distance to individual being observed decreased, observation quality code increased. (PDF) [file pone.0303312.s006.pdf]

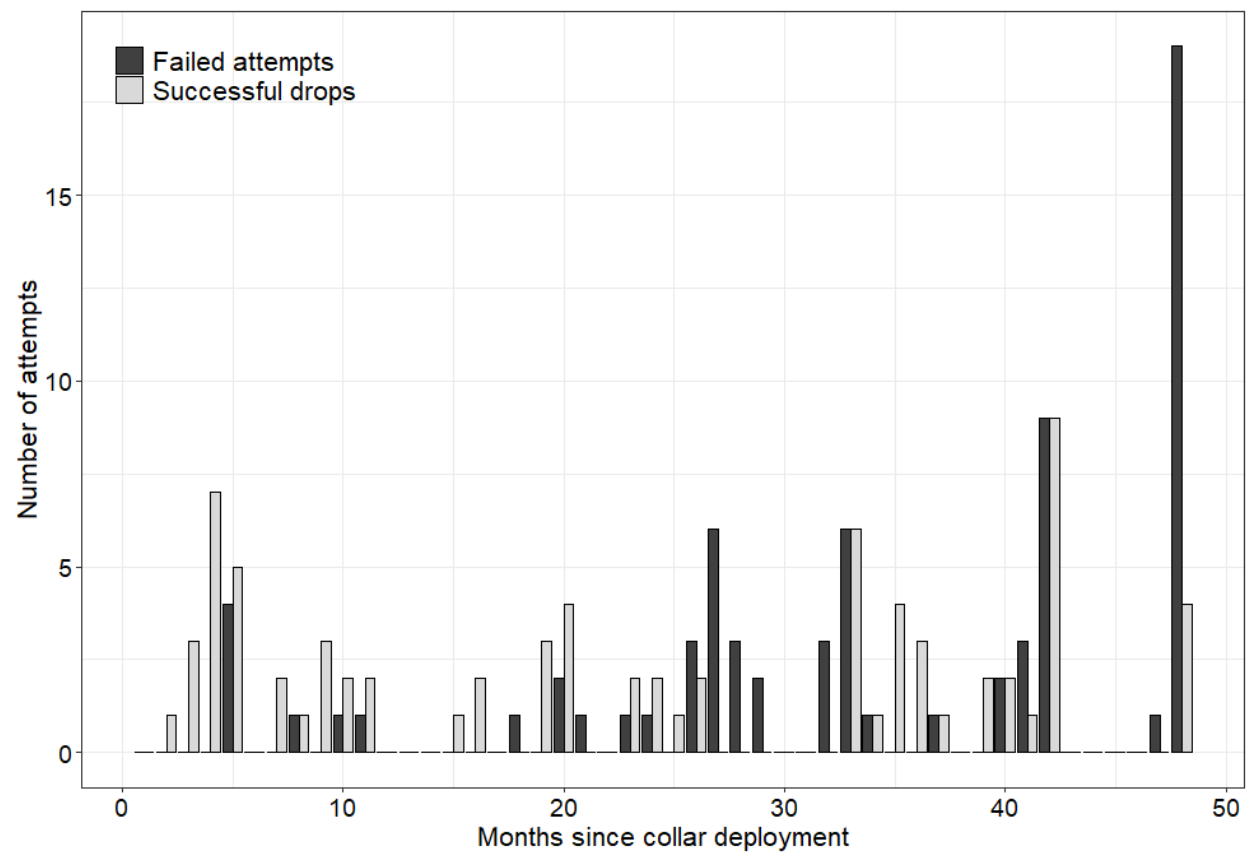

Supplement: S2 Fig — Attempts resulted in either a successful drop where the collar came off the animal, or a failed attempt where the collar failed to release after the drop command was sent. We made attempts to drop collars remotely at the end of the study primarily and not at different times throughout the study. Thus, we are unsure if collar drop offs may have failed sooner than at the time we tried them. (PDF) [file pone.0303312.s007.pdf]

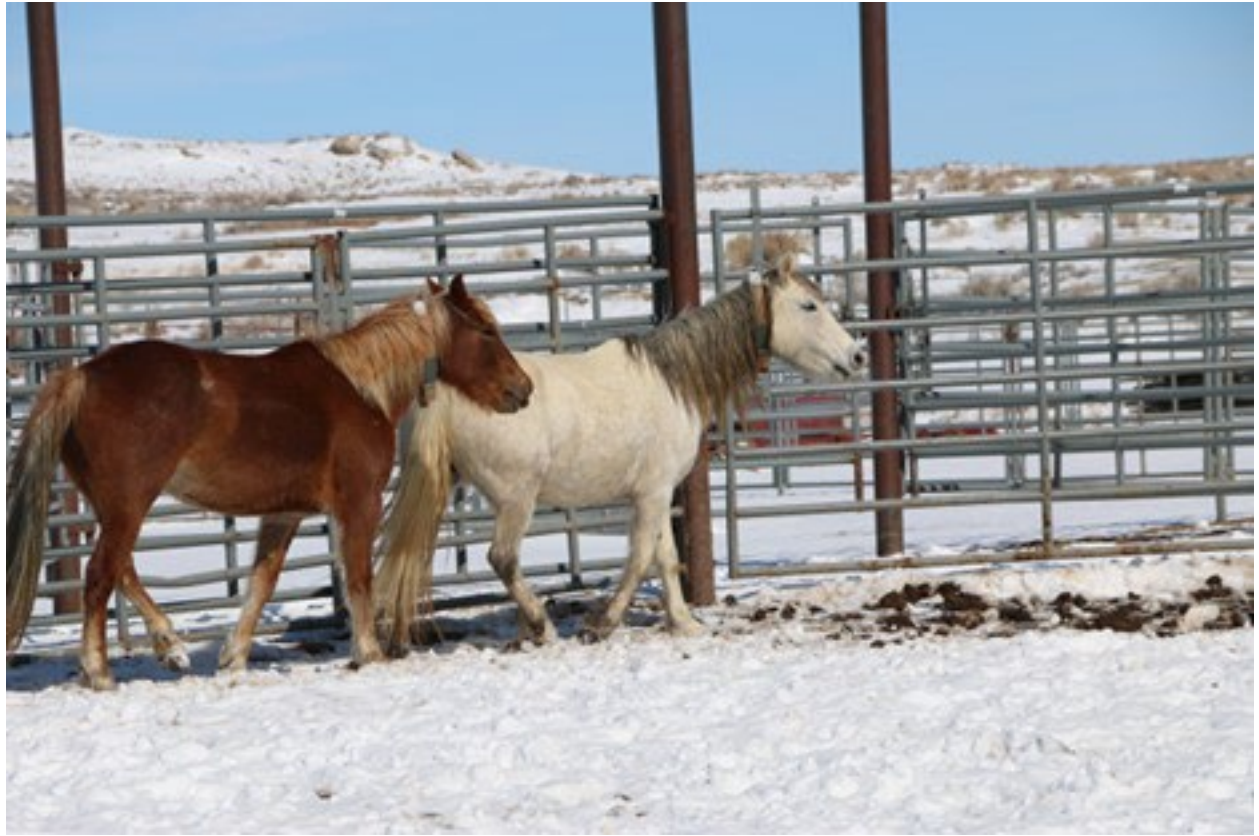

Supplement: S1 Photo — Photo credit K.A. Schoenecker. (PDF) [file pone.0303312.s009.pdf]

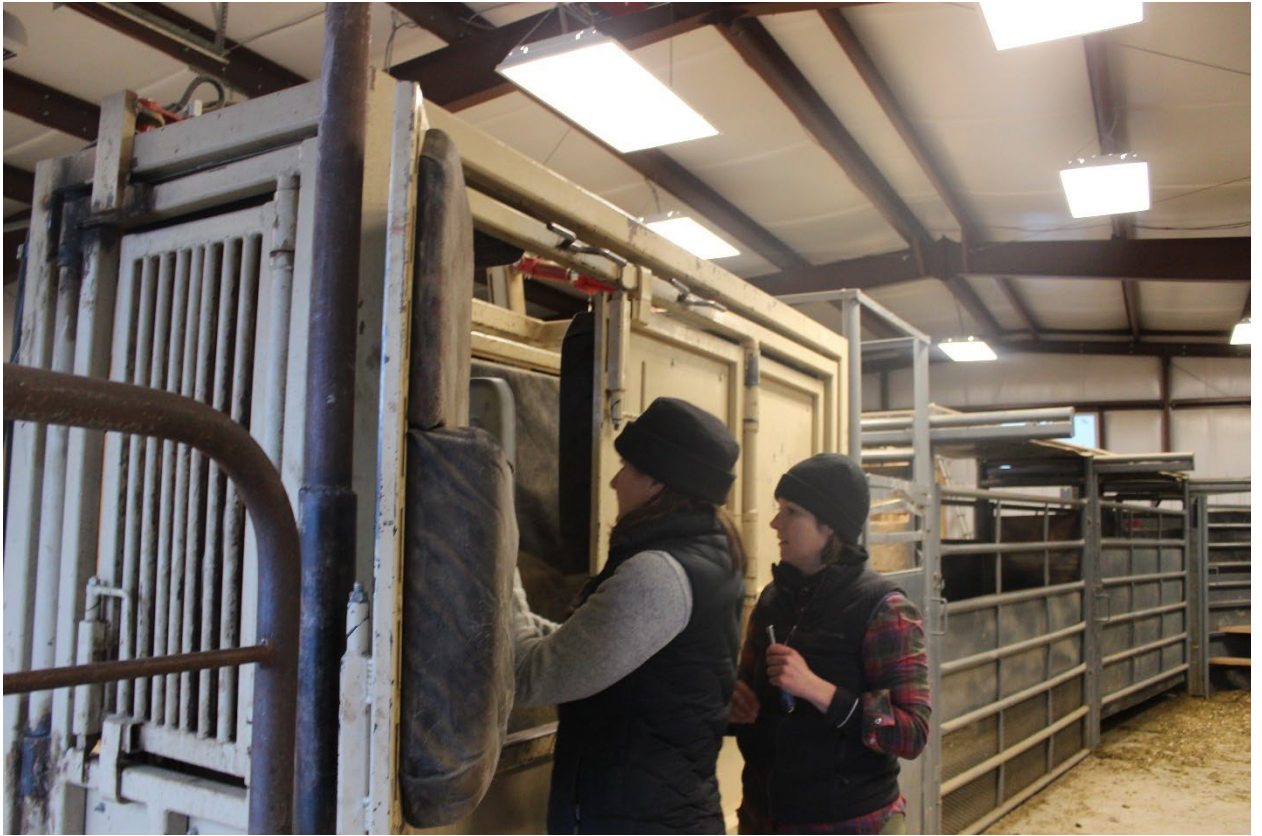

Supplement: S2 Photo — The individuals in this photograph have given written informed consent to publish this photo. (PDF) [file pone.0303312.s010.pdf]

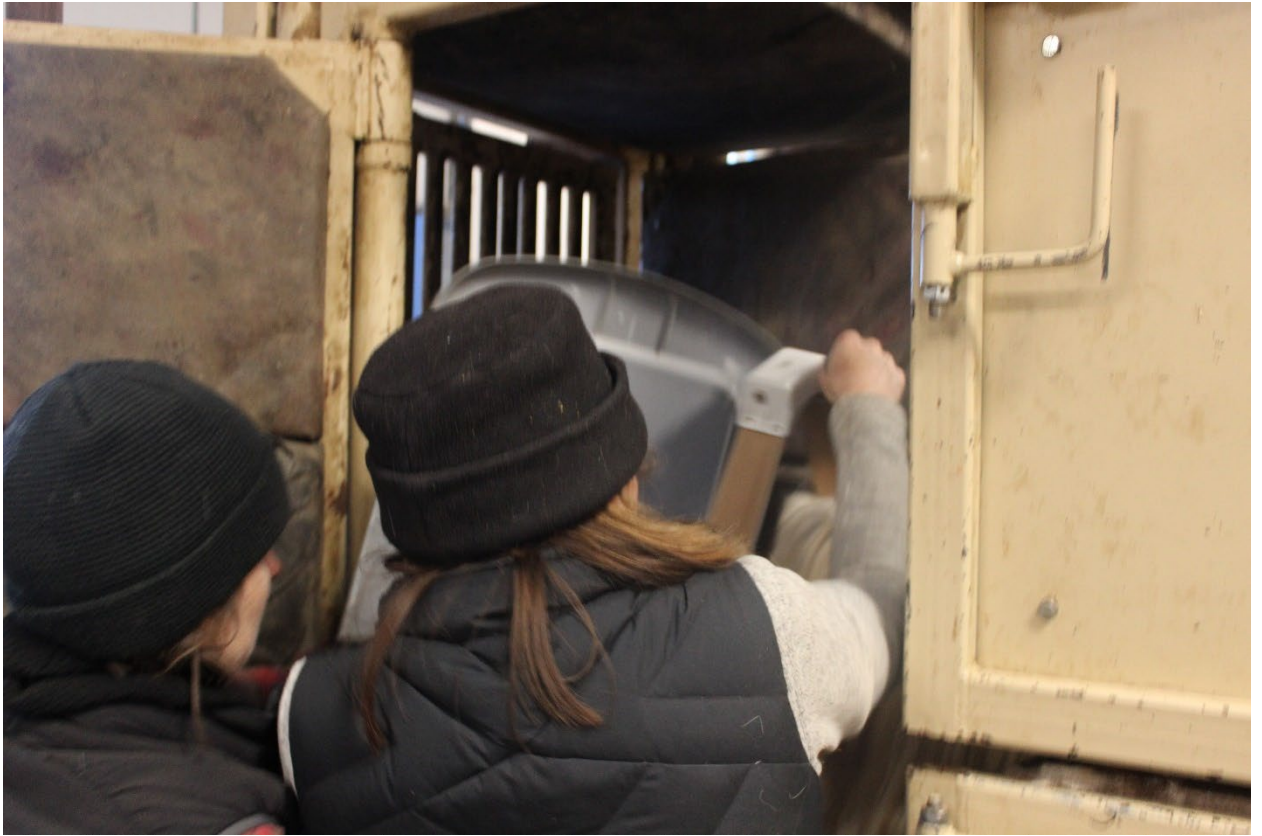

Supplement: S3 Photo — This visual block can also aid in keeping the mare calm, similar to the effect of blindfolds that are used for ungulate live captures. This collaring application technique was developed for a study of potential effects of collars on free-roaming equids, 2016–2020, USA. The individuals in this photograph have given written informed consent to publish this photo. (PDF) [file pone.0303312.s011.pdf]

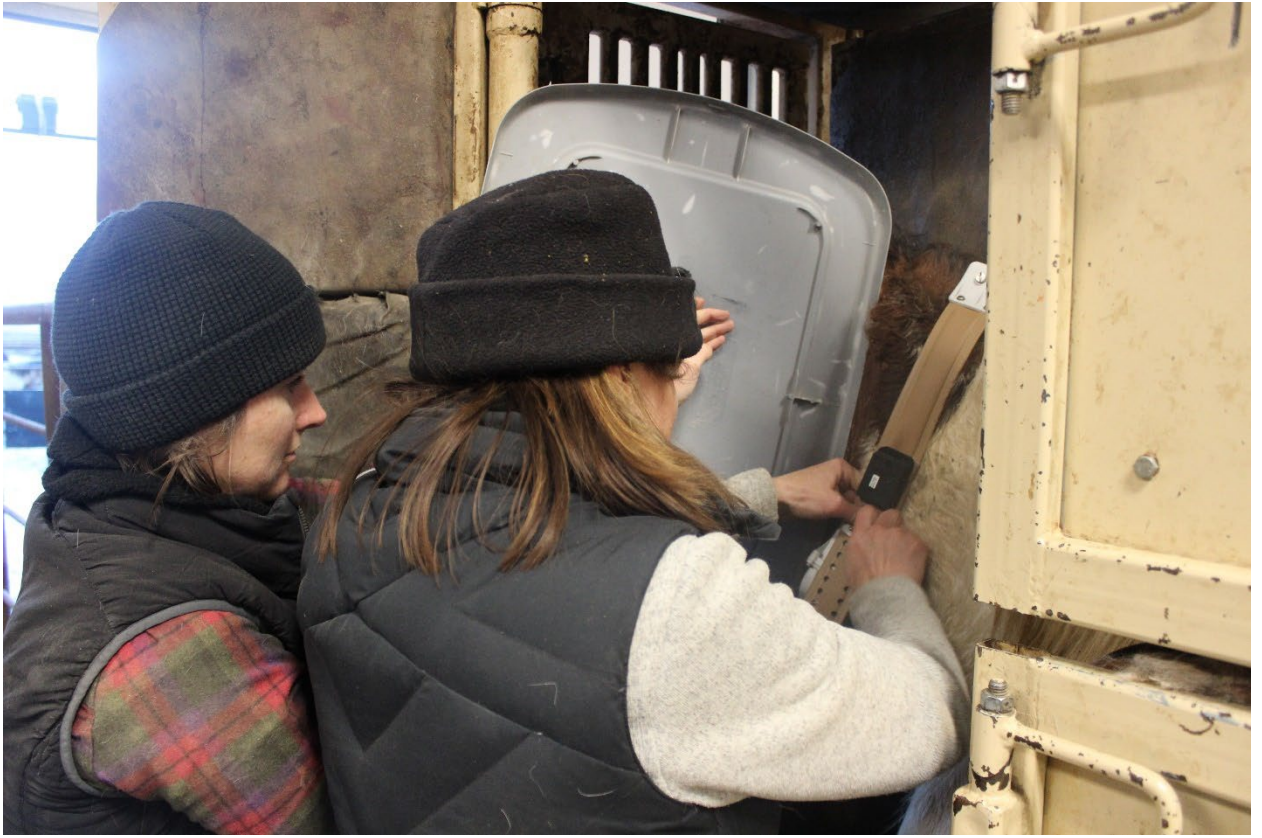

Supplement: S4 Photo — The black rectangle on the side of the collar is the remotely triggerable drop off mechanism. The individuals in this photograph have given written informed consent to publish this photo. (PDF) [file pone.0303312.s012.pdf]

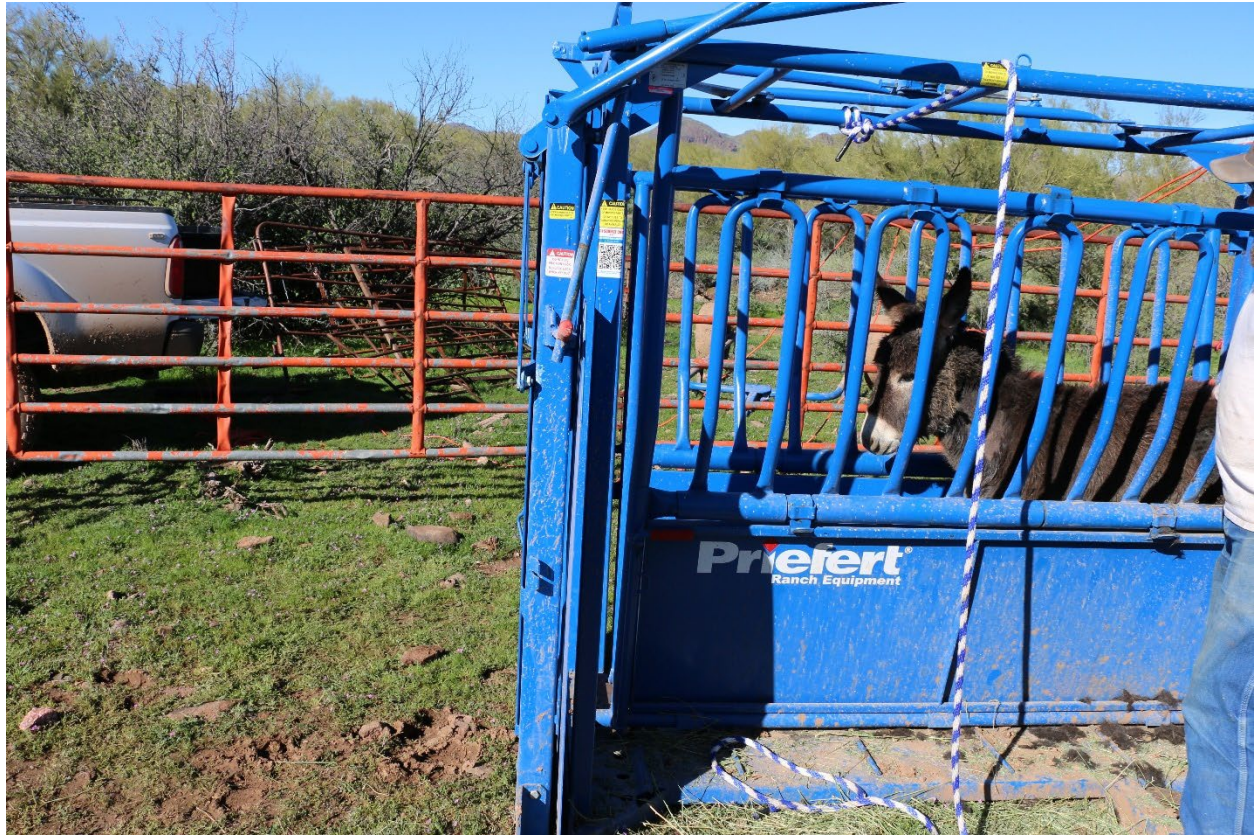

Supplement: S5 Photo — Photo credit: K.A. Schoenecker. (PDF) [file pone.0303312.s013.pdf]

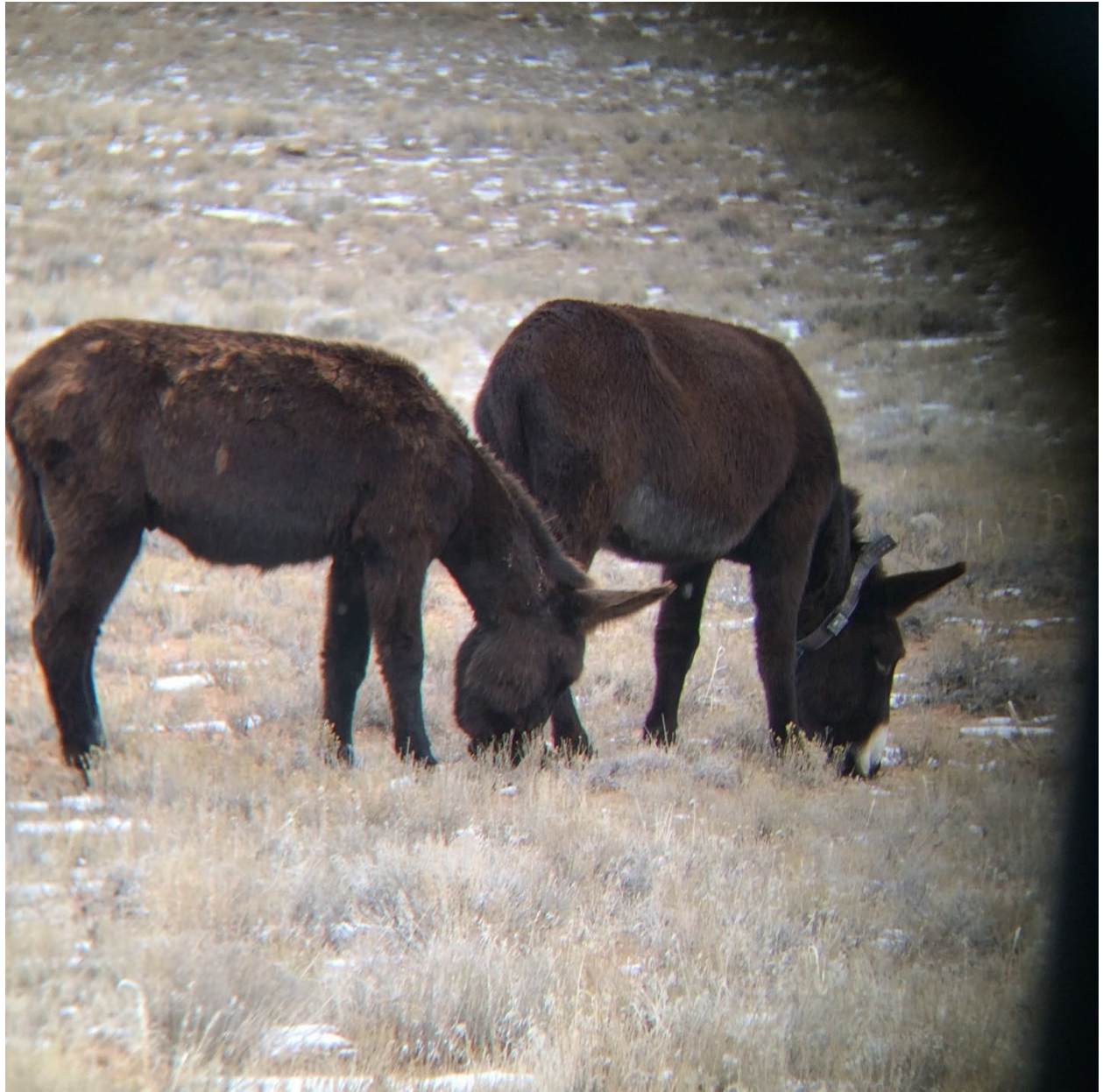

Supplement: S6 Photo — Photo credit: M.J. Cole. (PDF) [file pone.0303312.s014.pdf]

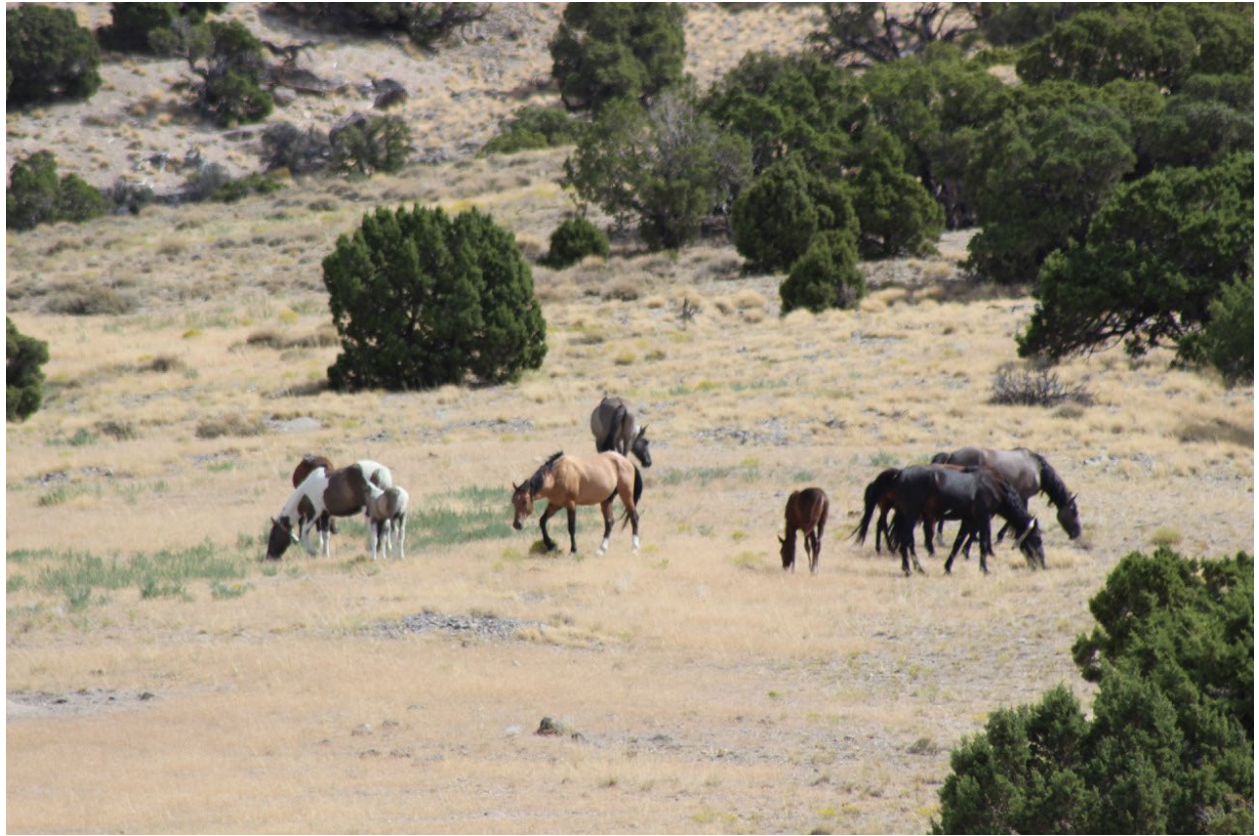

Supplement: S7 Photo — Photo credit: S.R.B. King. (PDF) [file pone.0303312.s015.pdf]

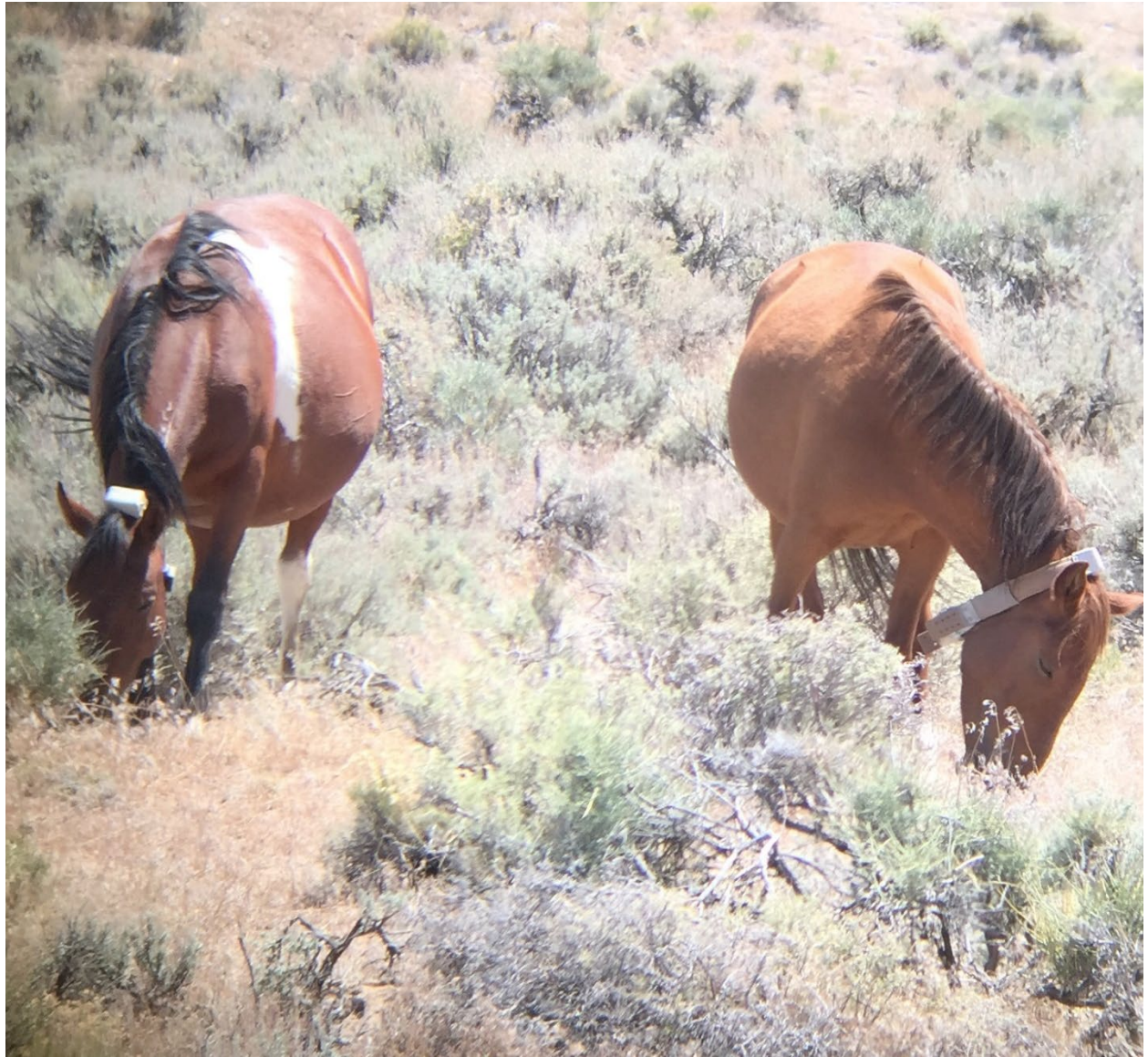

Supplement: S8 Photo — Photo credit: R. Fawbush. (PDF) [file pone.0303312.s016.pdf]

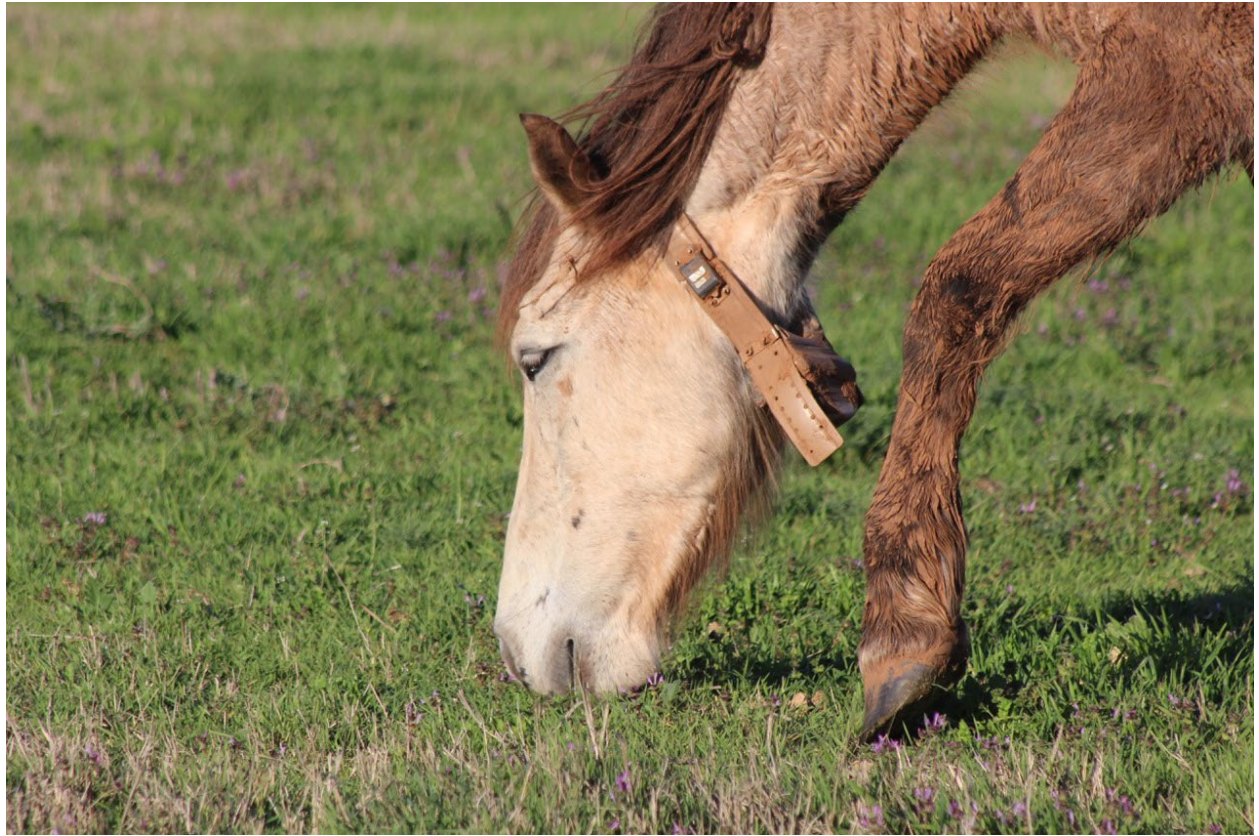

Supplement: S9 Photo — A remotely triggerable drop off mechanism is visible on the side of the collar. Photo credit: S.R.B. King. (PDF) [file pone.0303312.s017.pdf]
