# Supplementary material for: Effects of telemetry collars on two free-roaming feral equid species
Source: PLoS One. 2024 May 30;19(5):e0303312. doi: 10.1371/journal.pone.0303312 (PMC11139308; doi:10.1371/journal.pone.0303312)
Supplement: S1 Text — (PDF) [file pone.0303312.s008.pdf]

The purpose of this supplementary text is to provide a description of the methods that were used to fit GPS collars on horse mares and burro jennies. This document does not include methods for capture, immobilization, care or maintenance of horses or burros during gathers or bait or water trapping, while in captivity, or for any other handling procedures beyond those needed for fitting and removing a telemetry collar. All capture and handling for deploying and affixing GPS collars should follow established guidelines for animal welfare [1, 2]. Collar GPS communication systems should be tested prior to collar deployment.

Horse or burro restraint. It is possible to put the collar in place with minimal restraint depending on the disposition of the animal. Burros needed minimal restraint in a fly chute as long as they were prevented from moving excessively forward or backward, whereas horses were restrained in a padded squeeze chute for horse and human safety during our study. For collar fitting, the chute should not be squeezed too tightly because it changes the shape of the neck and can lead to poor fit; horses should be able to shift some in the chute and have all four feet on the floor. Patience while deploying collars is important to allow the animal to settle so correct fit can be achieved. Typically, horses and burros were calm while collars were being affixed because there was no discomfort in the process other than from being unnaturally close to humans. In our study it took approximately 5 minutes to place a collar in correct position, bolt the straps together, and tighten nuts on the collar (Table S5). Extra time may be needed to allow animals to release stress, then calm down, over several iterations. Burros sometimes sat down in the chute, so their chute time was extended. It is important to look again at the fit of the collar with minimal to no squeeze applied before releasing the animal from the chute.

Fitting of the collar. The collar should be turned on, VHF frequency checked, and remote drop-off device confirmed before being placed on a horse or burro. Fitting a collar on feral equids, particularly horses, is challenging because of the large difference in neck circumference with head up versus head down position [3]. That is, the shape of the neck when the head is near the ground grazing is narrower than when standing with head up, tall, alert, and with muscles engaged. For this reason, collars are placed directly behind the ears at the top of the neck because this area of the neck changes the least in different positions. Collars should be affixed snugly when the head is up, so there is not excess movement when the head is down. Burros have less change in their neck size during grazing, so their collars can be fitted more loosely than for horses. Collar fitting for burros follows the guidelines for other ungulates [4]; two flat fingers of an average adult person should fit under the collar for burros. Horse and burro collars should be flush with the neck to reduce opportunities for the collar to rub. Shape of equid collars should be oval or tear-drop to match the shape of their neck, as opposed to round. The edges of collar material should be rounded, not sharp.

After collar fitting, observe the animal for up to 1-2 days in the corral before it is released to the range. Watch it walk out of the chute. If collars on burros are too tight, they will stand mostly immobile in the holding pen and move around very little. They are unlikely to forage. If this occurs the collar is too tight and needs to be loosened. Due to horses tending to hold their heads high and tight in the chute, we found that often collars that seemed to be fitted snugly in the chute were loose when the horse was released to a corral. Thus, some horses may need to go through the sorting chute a second time to tighten the collar by one hole. This was common in our study.

Monitoring. Animals wearing a collar should be visually observed in the field at regular intervals (~4-8 weeks) to check for any irritation caused by the collar and to be sure the collar is in correct position. Monitoring is also useful to confirm that the collar is functioning properly. Collars should be equipped with a remotely triggerable drop-off mechanism to remove a collar in wrong position if needed, and a timed drop off that is scheduled to deploy before the end of the expected collar battery life.

Collar removal. Collars can either be 1) manually removed at the end of the study by recapturing the individual, 2) released with a timed release drop off mechanism in which the date is pre-set when the collar is deployed. Collars using an Iridium satellite link can be 3) triggered to drop off remotely, or 4) a separate unit with ultra-high frequency (UHF) communication can be attached to the collar to remove it on command.

Potential risks can be mitigated with proper fit. The potential risks involved in deploying radio collars on equids can be minimized through choosing the best collar design available for equids, correct fitting of the collar, and monitoring of the collared animal so the collar can be removed if needed. Whenever collars are deployed they should be fitted by experienced personnel who can attach the collar quickly but proficiently to minimize handling stress on the animal. Animals should be observed in the corral or another captive setting after 12-24 hours to be sure fit is correct before releasing them back to the range. Horses and burros should not wear collars if they are remaining for longer periods (i.e., >1 week) in a holding facility because the collar may be moved out of correct position while the animal is accessing feeding troughs (if animals need to

place their head through rungs or bars to access forage) or due to higher interaction rates while in close confinement with conspecifics. In this situation there is a higher likelihood the collar could be pulled into a wrong position or over the ears.

References

1.Bureau of Land Management (BLM). Wild horse and burro comprehensive animal welfare program. Permanent Instructional Memorandum 2021-002, U.S. Department of Interior, Bureau of Land Management, Washington, DC. 2020.

2. Cox, R.J., Nol, P., Ellis, C.K., & Palmer, M.V. Research with agricultural animals and wildlife. Institute for Laboratory Animal Research Journal 2019; 60:66-73.

3. Schoenecker, K.A., S.R.B. King, and G.C. Collins. Testing fit and wear of radio collars on feral horses in a captive setting. Human–Wildlife Interactions. 2020; 14:73-86.

4. Silvy, N.J. The Wildlife Techniques Manual. John Hopkins University Press and The Wildlife Society. 2020.
